# The Potential of Nutraceutical Supplementation in Counteracting Cancer Development and Progression: A Pathophysiological Perspective

**DOI:** 10.3390/nu17142354

**Published:** 2025-07-18

**Authors:** Carmen Altomare, Roberta Macrì, Maria Serra, Sara Ussia, Giovanna Ritorto, Jessica Maiuolo, Carolina Muscoli, Enzo Perri, Vincenzo Mollace

**Affiliations:** 1 Pharmacology Laboratory, Institute of Research for Food Safety and Health IRC-FSH, Department of Health Sciences, University Magna Graecia of Catanzaro, 88100 Catanzaro, Italy; carmen.altomare@studenti.unicz.it (C.A.); saraussia1598@gmail.com (S.U.); giovanna.ritorto@studenti.unicz.it (G.R.); muscoli@unicz.it (C.M.); mollace@libero.it (V.M.); 2 Laboratory of Pharmaceutical Biology, IRC-FSH Department of Health Sciences, University “Magna Græcia” of Catanzaro, 88100 Catanzaro, Italy; maiuolo@unicz.it; 3 Research Centre for Olive, Fruit and Citrus Crops, Council for Agricultural Research and Economics, 87036 Rende, Italy; enzo.perri@crea.gov.it

**Keywords:** Mediterranean Diet, nutraceuticals, dietary polyphenols, antioxidant compounds, oxidative stress, inflammation, cancer prevention, health-promoting phytochemicals

## Abstract

Cancer is a major cause of morbidity and mortality across the globe, with a substantial increase in cases anticipated over the next few decades. Given the constraints and adverse effects associated with standard cancer therapies, the contribution of diet and nutraceuticals to cancer prevention and treatment is receiving increased scrutiny. A diet rich in plant-based foods, extra virgin olive oil (EVOO), and bioactive compounds, including the Mediterranean Diet, has been associated with reduced cancer risk and improved treatment outcomes. This review aims to explore the complex mechanisms of the MedDiet and nutraceuticals (polyphenols, flavonoids, terpenoids) in cancer prevention, to determine their potential as cancer treatment adjuvants. Promising results show that key compounds such as bergamot polyphenolic fraction (BPF), cynaropicrin, oleuropein, quercetin, resveratrol, and serotonin can modulate oxidative stress, inflammation, the tumor microenvironment, the cell cycle, and drug resistance. A significant observation is that many of these substances demonstrate dual dose-dependent activity; they function as antioxidants in healthy cells but induce pro-oxidant and pro-apoptotic effects in cancerous cells. Their ability to boost chemotherapy’s effectiveness and safety while lessening side effects and offering combined advantages is also explored. To summarize, this review suggests that the Mediterranean Diet and nutraceutical supplements may help prevent and manage cancer, but more research is needed to confirm their benefits.

## 1. Introduction

Cancer is the second leading cause of death globally, trailing only heart disease. According to the International Agency for Research on Cancer (IARC), there were about 20 million new cancer diagnoses and 9.7 million deaths globally. By 2050, annual cases could hit 35 million, assuming current incidence levels persist [1]. Several factors, including lifestyle, daily routines, and diet, contribute to the rising cancer rates [2]. Epidemiological and clinical research shows a link between nutrition and the development or progression of various cancers (colon, breast, and prostate), classifying them as diet related [3]. The beneficial effects of a healthy lifestyle, and its correlation with the Mediterranean Diet’s (MedDiet) wide range of health benefits, are supported by several studies [4], including reduced risk of cancer and cardiovascular disease (CVD), reduced metabolic risk of diabetes mellitus (DM), obesity, and metabolic syndrome (MS), increased longevity, and improved cognitive function. Furthermore, the MedDiet is linked to a healthy gut microbiome, impacting cancer development and treatment.

The hallmark of cancer is the conversion of some normal cells into malignant neoplastic cells [5].

Therefore, uncontrolled proliferation, evasion of growth suppressors and the immune system, angiogenesis, metastasis, and metabolic reprogramming are the main characteristics [6]. Cellular and subcellular microenvironments and genetic instabilities are key to this multistep process. Malfunction of the core cell cycle leads to unstable deoxyribonucleic acid (DNA) and mutations, furthering tumorigenesis [7]. The high number of cancer cases urgently requires new treatment approaches to address the weaknesses of traditional methods like surgery, hormone replacement therapy (HRT), chemotherapy, and radiation, which often cause drug resistance and harmful side effects [8]. Because they boost tumor response to treatments and decrease systemic toxicity, the wide array of natural compounds in the MedDiet are a potential cancer-fighting strategy [9]. This review aims to highlight the effects of natural compounds, as nutraceuticals, in the management of cancer therapy. Due to their proven antioxidant and inflammatory properties, as well as their ability to modulate the cell cycle, natural compounds—in particular extracts from a selection of foods characteristic of the MedDiet, such as *Citrus bergamia* Risso & Poiteau, *Cynara cardunculus* L., *Olea Europaea* L., foods rich in quercetin, resveratrol (RV), as well as Mediterranean plants such as *Ferula communis* L.—may be relevant for synergism with chemotherapeutic drugs while reducing their related toxic effects.

### 1.1. Complexities of Cancer Pathogenesis

Cancer development is a complex process influenced by various factors, leading to diverse clinical presentations, and various factors contribute to its pathogenesis, each a potential therapeutic target (e.g., chemicals, viruses, genetic, and non-genetic factors).

Several studies have shown that carcinogenesis is related to somatic mutations of stem cells, which generate a subpopulation of cancer stem cells (CSCs) with self-renewal capabilities, producing daughter cells with highly tumorigenic and invasive potential, originating primary tumors [10]. Evidence suggests that epigenetic mutations can provide tumor cells with a reproductive advantage over normal cells, leading to impaired tumor suppression and uncontrolled tumor growth [11]. However, the inherent adaptability of healthy cells, allowing epigenetic and phenotypic shifts leading to CSC formation, is pivotal in cancer development [12]. Oncogene and tumor suppressor alterations in this scenario accelerate cell growth and may cause a more aggressive metastatic phenotype.

The occurrence of Rat sarcoma virus (RAS) and its family genes, Kirsten rat sarcoma viral oncogene homolog (KRAS) (*KRAS4A* and *KRAS4B*), Neuroblastoma RAS viral oncogene homolog (NRAS), or Harvey Rat sarcoma virus (HRAS) [13] are among the most dysregulated protooncogenes and best characterized. Oncogenic activation of RAS results in ligand-independent hyperstimulation of downstream signaling cascades, including the extracellular signal-regulated kinase 1/2 (ERK1/2) [14] and phosphatidylinositol 3-kinase (PI3K) promoting cell growth, cell cycle entry, cell survival, and cell adhesion. Moreover, mutations in KRAS lead to chemoresistance mechanisms [15]. The function of tumor protein p53 (p53), a tumor suppressor, is widely understood. p53 gene mutations disrupt the normal response to DNA damage, blocking apoptosis and cell cycle arrest, thus causing abnormal cell division. The development of cancer is linked to the epithelial–mesenchymal transition (EMT) and a metabolic shift from glycolysis to oxidative phosphorylation [16]. Epithelial cells undergoing EMT lose apical-basal polarity and cell–cell junctions, gaining mesenchymal characteristics, such as invasion and migration. In addition, cells develop stem cell characteristics and become resistant to chemotherapy [17]. Signaling pathways to activate and maintain EMT include notch signaling pathway (NOTCH), wingless-related integration site (Wnt), glycogen synthase kinase 3 beta (GSK-3β), and transforming growth factor beta (TGF-β) [18]. These pathways lead to loss of cell–cell adhesion and increased migratory cell ability by activating transcription factors including Slug, Twist, Snail, or Zinc finger E-box binding homeobox 1 and Zinc finger E-box binding homeobox 2 (ZEB1/2) [19], which in turn repress epithelial markers such as E-cadherin. Cancer cells metabolism shift from production energy by glycolysis to oxidative phosphorylation, mediated by pathways such as PI3K/Akt/mTOR and AMP-activated protein kinase (AMPK) [20,21]. This network encompasses cancerous and non-cancerous host cells, alongside the modified extracellular matrix (ECM) and vascularization. Tumor cell invasion is fueled by the tumor microenvironment (TME), a key factor in tumor development and resistance to treatment. Its inflammatory, hypoxic nature drives adaptive pathways, enzyme overexpression, high adenosine triphosphate (ATP), and low pH, resulting in acidity [22]. The TME includes immune cells from both innate and adaptative systems, endothelial cells (ECs), cancer-associated fibroblasts (CAFs), pericytes, adipocytes, and other cell types that vary by tissue. In the TME, there are also myeloid-derived suppressor cells (MDSCs), tumor-associated macrophages (TAMs), and regulatory T-cells (Tregs), which secrete immunosuppressive molecules such as transforming growth factor beta (TGF-β) or modulating immune checkpoint [23]. Furthermore, the tumor tissue’s commensal microbiome is a key TME component, affecting tumor development and growth. The distinct intratumoral and intracellular microbial composition of different tumor types allows for the correlation of microbial metabolic pathways and drug treatment characteristics. Moreover, dysbiosis can trigger a persistent inflammatory immune response, via the microbial activation of nuclear factor kappa-light-chain-enhancer of activated B cells (NF-κβ), a cancer-linked inflammation regulator, thus promoting tumor growth [24].

### 1.2. Nutrition and Nutraceutical Compounds in Cancer Risk Modulation

Diet, nutrition, and lifestyle are correlated with cancer incidence, progression, and treatment response in some epidemiological studies. Age-related cardiovascular and metabolic diseases are also linked, according to scientific evidence. Human and animal studies demonstrate a link between diet, epigenetic changes, and cancer risk. It is possible to assert that lifestyle and diet, and particularly the MedDiet model, play a key role as a modulator of cancer risk. It represents not only a diet but also a lifestyle model in the Mediterranean area, where the incidence of several types of cancer is lower than in other areas [25]. Such incidence is associated with a potential protective role of the MedDiet on human health [26]. The plan emphasizes plenty of vegetables, fruits, legumes, whole grains, and nuts. The diet features extra virgin olive oil (EVOO) as its main fat, plus regular fish, moderate animal protein, and red wine.

**Figure 1:**
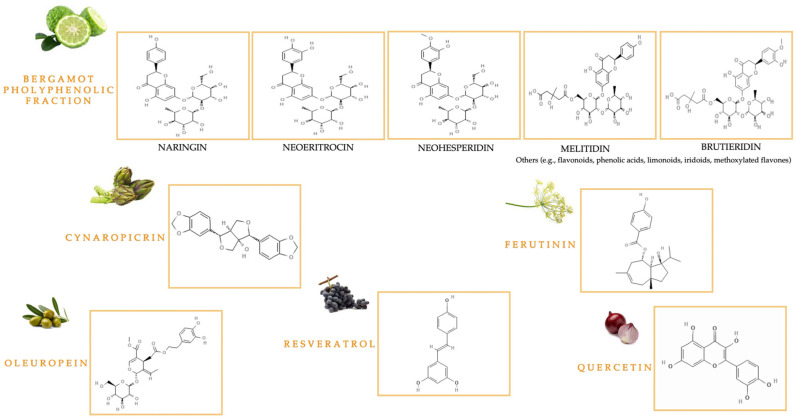
Diagrams of molecular structures.

The MedDiet has a high intake of B vitamins (vitamins B1, B2, niacin, B6, folate or B12), antioxidant vitamins (vitamins E and C), minerals (iron, selenium, phosphorus, and potassium), and fiber. A variety of nutraceuticals included in the MedDiet have shown an inverse association with CVD, metabolic disease, neurodegenerative disease, and various types of cancer, both directly and through epigenetic mechanisms [27]. Strict adherence to the MedDiet is associated with a lower risk of multiple diseases thanks to phytochemicals (“non-nutrient” components) present in its constituent foods, such as polyphenols, phytosterols, carotenoids, monounsaturated fatty acids (MUFAs) and polyunsaturated fatty acids (PUFAs) [28]. Specifically, polyphenols, water-soluble phenylpropanoids, contain at least one aromatic ring and a benzene ring with hydroxyl substituents. Plants synthesize these secondary metabolites to cope with stress like pathogen attacks, ultraviolet radiation (UV) radiation, or harsh weather. They are classified as: (i) flavonoids, characterized by the formula C6-C3-C6, among which there are flavonols, flavanols, flavones, flavanones, isoflavones, and anthocyanins; (ii) non-flavonoids, characterized by only one phenolic ring, including phenolic acids (hydroxybenzoic acids such as vanillic acid and gallic acid) and cinnamic acids (ferulic and caffeic acids), lignans, tannins, and stilbenes, including RV [29]. Foods contain various polyphenols such as flavan-3-ols, isoflavones, flavonols, flavanones, flavones, anthocyanins, and stilbenes, impacting color, taste, smell, bitterness, astringency, and resistance to oxidation. The range of polyphenol levels is widely conditioned by environmental conditions and soil type [30]. Studies demonstrate the relationship between the structural chemical conformation of polyphenols and their biological actions, affecting bioavailability and absorption [31]. In fact, the complex nature of polyphenols results in poor small intestinal absorption. Following absorption, liver enzymes (glucuronidation, sulfonation, methylation) modify phenolic substances before systemic circulation and tissue distribution. The inability of complex phenols to be absorbed leads to their biotransformation by the gut microbiota into smaller, bioavailable derivatives [32]. Epidemiological studies suggest that sufficient polyphenol intake, from food or supplements, may reduce oxidative stress, chronic inflammation, and cancer risk [33]. Moreover, increased polyphenol intake enhances cardiometabolic health via anti-inflammatory, antioxidant, vasodilatory, high-density lipoprotein (HDL)-raising actions, and by inhibiting low density lipoprotein (LDL) oxidation [34]. Polyphenols have been shown to reduce blood glucose levels by mechanisms including the inhibition of glucose uptake in the gut and other tissues [35]. Indeed, epidemiological studies also demonstrate that polyphenols protect against Metabolic Syndrome, a combination of hypertension, dyslipidemia, insulin resistance, and central obesity that contributes to cardiovascular and metabolic diseases [36]. Polyphenols can restore redox balance [37], impacting arachidonic acid metabolism through the inhibition of various enzymes, such as phospholipase A2 (PLA2), lipoxygenase (LOX), and cyclooxygenases (COX-1 and COX-2); consequently, there is a reduction in the release of prostaglandins, thromboxanes, leukotrienes, and other mediators of inflammation [38]. Metal ion chelation mechanisms enable these molecules to exert their chemoprotective effects, including cell cycle arrest induction, pro-apoptotic process induction, and immune system regulation [39]. Polyphenols have both estrogenic and antiestrogenic effects, impacting how anticancer drugs are processed in the body, thus reducing their toxicity [40]. In addition, some polyphenols have been shown to be able to disrupt the elongation of telomeric ends, which could have an important impact on cancer cell replication, and they are effective as anti-aging compounds [41]. In fact, the level of antioxidants in plasma depends on the consumption of antioxidant-rich foods and supplements, such as those containing polyphenols. Natural compounds, by modulating specific proteins, can regulate drug resistance, which occurs through several mechanisms, including enhanced DNA damage repair, invasiveness, altered drug efflux and metabolism, modification of drug targets and TME [42]. The important role of nutraceuticals in oncology has led to them being called onconutraceuticals [43]. In cancer treatment, their application focuses on discovering synergistic effects with anticancer drugs to improve treatment success, decrease side effects (e.g., cardiotoxicity, hepatotoxicity), and overcome drug resistance. Table 1 generated from the literature data, presents the principal biological properties of the key nutraceuticals of the MedDiet and Figure 1 shows the molecular diagrams of the main natural compounds discussed in this review.

#### 1.2.1. Bioactive Compounds from *Citrus bergamia* Risso & Poiteau and *Cynara cardunculus* L.

*Citrus bergamia* Risso & Poiteau, commonly referred to as “Bergamot”, is a Mediterranean citrus fruit, and is considered an endemic plant of the Calabria region. In fact, while its precise botanical and geographical origins are unknown, bergamot is cultivated along the Calabrian Ionian coast, between Bianco and Reggio Calabria [44]. This plant belongs to the *Rutaceae* family, *Esperidea* subfamily. Three varieties of bergamot are known—“*Fantastico*”, “*Femminello*”, and “*Castagnaro*”. The relevance of this plant stems from the biological role of herbal preparations, particularly BEO and BJ. BEO is a volatile oil obtained by scraping and cold pressing the fruit peel; it contains up to 93–96% volatile compounds (limonene, linalool, and linalyl acetate), while the remaining percentage is a variable fraction of non-volatile substances (pigments, waxes, coumarins, and psoralens) [45]. BJ is derived from the endocarp and pulp of bergamot fruits, featuring a distinctive composition of compounds also present in albedo, including flavonoids like naringin, neohesperidin, and neoeriocitrin, as well as C-glucoside, flavanone O-glycosides, and flavone O-glycosides such as rhoifolin 40-O-glucoside, neodiosmin, rhoifolin, and poncirin, along with furanocoumarins like bergapten and bergamottin. The major biologically active polyphenols from BJ were found in the bergamot polyphenolic fraction (BPF). High-dose toxic furanocoumarins are removed from the BJ through an alkalinization step in production [61]. The key attraction of bergamot is the diverse health benefits stemming from the pharmacological activity of its components. Furthermore, the recent circular economy approach has highlighted the value of bergamot waste, supporting its use in therapies and advancing scientific understanding [46,47].

*Cynara cardunculus* L., commonly referred to as “cardoon,” represents a perennial Mediterranean plant belonging to the *Cardueae tribe Cass*. (*Cynarae Less*.), *Asteraceae* family, which includes three botanical taxa, the artichoke (*C*. *cardunculus* L. var. *scolymus* L. Flowers), the perennial wild thistle (*C*. *cardunculus* L. var. *sylvestris* (Lamk) Flowers), and the domestic thistle (*C*. *cardunculus* L. var. *altilis* DC.). This Mediterranean plant thrives despite harsh conditions, including extreme temperature swings and poor soil [62]. The immature flower structure of the artichoke, encompassing the leaves (bracts) and upper receptacle, is what is edible. Due to its nutritional profile—high in minerals, vitamins, amino acids, proteins, and low in lipids—cardoon qualifies as a functional food. The roots and rhizomes are rich in inulin, a crucial carbohydrate contributing to its high nutritional value and supporting gut microbiota [49], while cardoon leaves are a source of antioxidants such as dicaffeoylquinic acids and luteolin. Inedible plant matter accounts for 80–85% of plant biomass, resulting in wasted bioactive substances. Thus, the byproduct use of non-food parts yields useful pharmacologically active molecules such as polyphenols and flavonoids. Chlorogenic acid (5-O-caffeoylquinic acid) and cynaropicrin, a sesquiterpene lactone, are responsible for cardoon’s bioactivity, including hepatoprotective, antioxidant, chemopreventive, antiobesity, and antimicrobial properties [48].

#### 1.2.2. Bioactive Polyphenolic Compounds in Olive Oil

 Olive Oil (OO) represents the most important product obtained from the evergreen tree in the Mediterranean basin *Olea Europaea* L., a key component of the MedDiet. The process uses only water and room temperature to crush the olives during malaxation, creating the product without chemical solvents. The biological activity of Olive Oil is largely due to its rich phytochemical composition, most notably the lipophilic fraction (95–97%) of MUFAs (e.g., oleic acid) and PUFAs (e.g., omega-3 and omega-6). The other fraction consists of tocopherols, phytosterols, squalene, and phenols including oleuropein, tyrosol, and hydroxytyrosol [63,64]. The European Food Safety Authority (EFSA) has determined that “phenols in Extra Virgin Olive Oil (EVOO) contribute to the protection of blood lipids from oxidative stress,” and this claim can be added to a product label when it contains at least 5 mg of hydroxytyrosol and its derivatives (e.g., oleuropein) per 20 g. Preclinical data indicates that oleuropein and hydroxytyrosol offer cardioprotection via mechanisms, including oxidative stress reduction, anti-inflammation, and homocysteine/cholesterol level modulation [50]. EVOO’s unique phenolic profile, shaped by factors like cultivar and processing, is vital to its role in preventing a range of diseases, from Metabolic Syndrome to cancer [51,52]. Furthermore, Olive Mill Wastewater (OMWW) a byproduct from industrial EVOO processing, contains a high concentration of bioactive polyphenolic compounds (e.g., oleuropein) [53].

#### 1.2.3. The Key Bioactive Role of Quercetin and Resveratrol

Quercetin and resveratrol, bioavailable polyphenols with antioxidant, anti-inflammatory, antiviral, and antiproliferative properties, are commonly found in foods characteristic of the MedDiet. Specifically, the flavonol quercetin (2-(3,4-dihydroxyphenyl)-3,5,7-trihydroxychromen-4-one) is prevalent in nature; key dietary sources include fruits and vegetables such as apples, berries, and onions [65]. Plants contain quercetin as glycosides (with attached sugars) or aglycones (without attached sugars), but the most common type, quercetin-3-O-glucoside, is hydrophilic and thus poorly absorbed. Consequently, quercetin demonstrates very low oral bioavailability. Extensive research focuses on this plant compound due to its effects on metabolic disorders, eye diseases, arthritis, and CVD [54,55].

Resveratrol is a stilbene and is produced by UV irradiation or fungal infection. However, antitumor, antioxidant, and anti-inflammatory activities are due to the trans-form of this bioactive compound [56], whereas there is currently only limited evidence for the cis- isomer [66]. RV is found in various nutritious foods, including grape skins and seeds, and is frequently detected in red wine, blueberries, cranberries, peanuts, and bilberries. The glycosylation of RV in food enhances its stability and bioavailability by preventing enzymatic oxidation [67]. The nutraceutical potential of quercetin and RV is being actively researched due to their effects on inflammation, gut microbiota, oxidative stress, and cancer [57,58].

#### 1.2.4. Bioactive Compounds from *Ferula communis* L.

The genus *Ferula communis* L. subsp. *communis* (Giant fennel) belongs to the *Apiaceae* family and is an herbaceous perennial plant containing latex. Central and South-West Asia, North Africa, and the Mediterranean region are home to approximately 180 species. Two distinct chemotypes have been identified, exhibiting different biological effects: a poisonous type with prenylated coumarins, and a non-poisonous type with sesquiterpene daucane esters. In addition, in plants of the genus *Ferula*, oleo-gum-resins are also found [68]. They are defined by many natural bioactive compounds, primarily from roots, leaves, and rhizomes, including sesquiterpenes, sesquiterpene lactones, flavonoids, coumarins (like ferulenol), coumarin esters (like ferulone A, B), and phytoestrogens (like ferutinin) [69]. The non-toxic chemotype’s phytoestrogens make it estrogenic—high extract concentrations can thus create a doubled ferutinin dose–response; unlike the other chemotype, which is toxic and causes severe hemorrhagic intoxication from the ingestion of its aerial parts leading to potentially lethal “ferulosis” [70]. The toxicity is due to the prenylated coumarin ferulenol; removing it makes the extracts non-toxic since the remaining prenylated coumarins are not toxic at their concentrations. Several *in vivo* and *in vitro* studies reported its pharmacological properties, including antidiabetic, antimicrobial, antiproliferative, and cytotoxic actions [59,60]. *In vitro* studies and preclinical models have shown that ferutinin can be considered a selective estrogen receptor modulator (SERM), but its mechanism of action is not totally clear compared with classical SERMs. Its action on estrogen receptors depends on cell type and concentration; it is an estrogen receptor alpha (ERα) agonist and may act as an estrogen receptor beta (ERβ) agonist or antagonist. Therefore, the effects of ferutinin on gene expression and cell signaling pathways within estrogen-sensitive cancers (e.g., ER-positive breast cancer (BC) and endometrial cancer (EC)) are dose-dependent [71]. Further studies of this typical Mediterranean plant need to explore the underlying mechanisms and potential pharmaceutical applications.

## 2. Biological Functions and Pathological Implications of Reactive Oxygen and Nitrogen Species

Reactive oxygen/nitrogen species (ROS and RNS) are unstable, thus highly reactive and partially reduced, oxygen- and nitrogen-derived molecules that include superoxide anion (O_2_^−^), hypochlorous acid (HOCl), hydroxyl radical, peroxyl radicals, nitric oxide, and peroxynitrite. The presence of a free electron on the oxygen atom allows oxygenated free radicals to react with sugars, proteins, and lipids; among free radicals, the superoxide anion is the most prevalent: oxidative phosphorylation in mitochondria, along with activated phagocytes, produces it via electron leakage from the electron transport chain to molecular oxygen [72].

Primarily, superoxide (O_2_^−^) will dismutate into oxygen and hydrogen peroxide (H_2_O_2_). Although superoxide is unreactive with DNA, H_2_O_2_, in the presence of iron or copper, can become highly reactive hydroxyl radicals (•OH) through the Fenton reaction. The generation of ROS, including this process and superoxide-derived ROS, is linked to various diseases, including cancer, cardiovascular diseases, and neurodegenerative disorders [73,74]. In hepatocytes and macrophages, ROS are produced during processes like aerobic respiration and inflammation. ROS signaling, regulated by molecules and post-translational modifications (PTMs), triggers specific cellular responses based on ROS generation’s nature and duration [75]. Cell differentiation and apoptosis, induced by ROS, contribute to physiological aging. RNS include nitric oxide and peroxynitrite: these molecules readily oxidize, thus damaging DNA and other biomolecules. Oxidative stress biomarkers are ROS-production enzymes such as nicotinamide adenine dinucleotide phosphate (NADPH) oxidase, myeloperoxidase, xanthine oxidase (XO), and endothelial nitric oxide synthase (eNOS). Well known biomarkers of ROS-induced chemical modifications are the advanced glycation end products (AGEs), the oxidized low-density lipoprotein (OxLDL), and malondialdehyde (MDA) [76]. Therefore, a crucial factor is keeping the body’s oxidative state balanced by generating and eliminating free radicals properly [77].

Indeed, high cellular ROS levels damage intracellular components like DNA, causing mutations and cell transformation [78]. Current findings suggest a link between ROS production and both internal and external influences. The electron transport chain within mitochondria is the primary endogenous source of ROS. High ROS levels cause DNA damage, oncogene activation, and tumor growth, whereas moderate levels induce cell death or senescence, thus suppressing tumor development [79].

Exogenous sources of ROS involve an unbalanced diet, anticancer therapy, radiation, smoking, drugs, and alcohol [80]. Chemotherapy drugs, including anthracyclines (doxorubicin (Dox), daunorubicin (Daun)), and camptothecins can cause various complications in the CV system due to ROS [81]. This is related to an increased lipid peroxidation and reduced levels of antioxidant molecules such as glutathione (GSH) [82]. It represents a non-enzymatic antioxidant, along with vitamins A, C, and E, and flavonoids. Enzymatic antioxidants include glutathione peroxidase (GPX), glutathione reductase (GRX), catalase (CAT), superoxide dismutase (SOD), superoxide reductase, thyroxine (TRX), and peroxiredoxin (PRX). The GSH system involves GSH, GR, glutathione transferase (GST), and GPX. Its deficiency or an altered GSH/GSSH ratio can lead to inflammation and tumor progression due to the occurrence of oxidative stress [83]. Dietary antioxidants are crucial, as evidenced by research on the diverse array of antioxidant systems [84,85]. By modulating oxidative stress, natural compounds can boost the effectiveness of therapies: the Mediterranean region, in this context, boasts an abundance of antioxidant-rich natural compounds [48,86].

### 2.1. Oxidative Stress in Cancer Development: The Role of Scavenger Enzymes

Increasing ROS release is observed during tumor development, activating mechanisms that drive tumor progression. This causes DNA damage and genetic instability, thus increasing cancer cell survival and resistance to cell death; consequently, cancer cells develop a more aggressive nature and capacity for metastasis. Additionally, high ROS levels trigger resistance mechanisms in tumor cells, which concurrently maintain pro-tumorigenic signaling. Furthermore, oxidative stress contributes to resistance against chemotherapy [87]. Therefore, altering the oxidized/reduced state ratio may offer an oncological treatment, linked to the antioxidant effects of various natural compounds [88]. Unbalanced oxidative stress levels lead to the activation of oncogenes and reduction in the activity of tumor suppressors. High ROS production in cancer can result in the inactivation of H_2_O_2_ scavenger enzymes like glutathione peroxidase (GPX), glutathione reductase (GRX), catalase (CAT), superoxide dismutase (SOD), superoxide reductase, thyroxine (TRX), and peroxiredoxin (PRX) and the tumor suppressor gene phosphatase and tensin homolog (PTEN), a negative regulator of the PI3K/AKT signaling pathway, as well as the oxidation and inactivation of many other protein tyrosine phosphatases (PTPs) such as tyrosine-protein phosphatase non-receptor type 1 (PTP1B) [89]. PTEN oxidation and inactivation results in cell survival through the activation of PI3K and protein kinase B (Akt) pathways [90]. Akt is involved in cell survival through phosphorylation and the inactivation of its target proteins, including the pro-apoptotic transcription factors: forkhead box transcription factors (FOXO), BCL2 associated agonist of cell death (BAD), BCL2 associated X apoptosis regulator (BAX), and B-cell lymphoma 2 (Bcl-2)-like protein 11 (BCL2L11) [91]. Additionally, several cancers, including esophageal squamous cell carcinomas, stomach adenocarcinomas, and colorectal carcinomas (CRCs), show increased expression of genes that code for SOD proteins [92]. Tumor pathogenesis is associated with mutations in transcription factors, such as nuclear erythroid 2-related factor (Nrf2), and tumor suppressor genes, such as p53 [93]. In fact, under physiological conditions, the expression and activity of the transcription factor Nrf2 are strictly degraded by Kelch-like ECH-associated protein 1 (KEAP1), an adaptor of Cullin 3-based E3 ubiquitin ligase [94]. Cytosolic KEAP1 binding to Nrf2 triggers Nrf2 ubiquitination and proteasomal degradation; consequently, Nrf2 levels remain low under normal conditions. Oxidative stress causes the oxidation of thiol groups within KEAP1 (e.g., S-S cross-links) and this conformational change causes the release of Nrf2 in the cytosol, with its subsequent translocation to the nucleus, where Nrf2 activates antioxidant response element (ARE), which regulates antioxidant enzymes such as NAD(P)H quinone oxidoreductase (NQO1), glutathione S-transferases (GSTs), thioredoxin reductase-1 (TXNRD1), thioredoxin, ferritin, glutamate-cysteine ligase, and heme oxygenase-1 (HO-1), depending upon the binding site present in the promoter region. Nrf2 has been reported to be active and overexpressed in cancer, promoting tumor progression and cancer cell survival [95]. Its activation can also be mediated by other signal transduction pathways such as AMPK, extracellular signal-regulated kinase (ERK), c-Jun amino-terminal kinase (JNK), and PI3K/AKT [96].

### 2.2. Pathways Involved in Oxidative Stress-Induced Tumorigenesis

Tumorigenesis frequently involves the constitutive activation of the Nrf2 pathway, as shown in various studies. This activation arises from mechanisms including oncogene (KRAS, B-Raf proto-oncogene serine/threonine kinase (BRAF), cellular myelocytomatosis oncogene (c-myc) driven transcription, KEAP1 epigenetic suppression, or KEAP1 somatic mutations disrupting Nrf2 binding [97]. In addition, growth factors and KRAS-stimulated pathways have been shown to activate MAPK/ERK 1/2 in cancer, a protein kinase that is a member of the mitogen-activated protein kinase (MAPK) family resulting in increased cell proliferation [98]. Estrogen metabolism generates H_2_O_2_, which triggers the ERK1/2 signaling pathway in various cancers, such as BC; cancer cell motility and anchorage-independent growth are also impacted by the latter. Mitochondrial dysfunction-induced ROS production increase promotes NF-κB upregulation [99] which induces the up-regulation of epidermal growth factor receptor (EGFR) and its ligands through polycystin 1, transient receptor potential channel interacting (PKD1)-mediated signaling. PKD-family serine/threonine kinases act downstream of protein kinase C (PKC) and other signaling pathways, including those of Src and Abl, play a key role in detoxification from ROS and the transcription of anti-apoptotic genes [100]. The promotion of tumor progression by PKD1 involves supporting mechanisms for cancer cell survival. This includes activating ERK1/2 and regulating the JNK pathway, with effects varying based on the cellular environment [101]. Low H_2_O_2_ levels can trigger NF-κB, an oxidative stress detector; this can trigger the production of anti-apoptotic genes and pro-cancer cytokines like interleukin-6 (IL-6) and tumor necrosis factor alpha (TNF-α) and may activate or suppress MAPK phosphorylation. Moreover, blocking NF-κB may overcome drug resistance, a key factor in the development of chemo-resistance [102]. Reduced ROS activates hypoxia-inducible factor 1-alpha (HIF1-α), leading to the expression of genes essential for cancer growth, such as the vascular endothelial growth factor (VEGF) and its receptors; high ROS levels can directly cause oxidative DNA damage [103] and interfere with the function of epigenetic modifiers, such as DNA methyltransferases (DNMTs) and histone deacetylases (HDACs), resulting in both the hypomethylation and hypermethylation of DNA. Oxidative DNA damage causes alterations such as base pair deletions, insertions, mutations, and double-strand breaks (DSBs), forming mutagenic 8-oxo-2′-deoxyguanosine (8-oxodG), which can accumulate and cause cancer. Furthermore, telomere 8-oxo-G accumulation affects length and impairs end-capping maintenance [104]. ROS have a direct impact on the ability of transcription factors (TFs) to bind DNA: increased localization of Fos/Jun DNA-binding redox factor-1 (Ref-1) protein and ataxia-telangiectasia mutated (ATM) serine/threonine kinase—key to DNA repair—occurs in the nucleus. High ROS levels promote cancer metastasis by suppressing natural killer (NK) cell activity [105] and can regulate macrophages recruitment and cancer cell invasion. In fact, ROS contribute to the acquisition of metastatic potential by cancer cells through EMT and the expression of TF Snail, which downregulates the expression of epithelial cadherin (E-cadherin) and promotes the expression of neural cadherin (N-cadherin) and vimentin. Excessive levels of ROS can lead to the activation of intrinsic and extrinsic apoptosis; in particular, mitochondrial excess ROS levels play a key role in initiating intrinsic apoptosis. High ROS levels lead to the release of cytochrome C (cyt C) into the cytosol, where it engages the apoptotic protease-activating factor 1 (APAF1), followed by the formation of the apoptosome and the activation of caspase-9 [106]. ROS can activate the extrinsic apoptotic pathway by modulating death receptors at the transmembrane level, including Fas (CD95), tumor necrosis factor receptor 1 (TNFR-1/TNF-α receptor) and tumor necrosis factor-related apoptosis-inducing ligand receptors (TRAIL-R1/2). Activated receptors, in the cytosol, recruit adaptor proteins (e.g., Fas-Associated Death Domain Protein (FADD)) and procaspases-8/10, forming death-inducing signaling complexes (DISCs) that activate caspases-8 and -10. Direct activation of effector caspases -3, -6, and -7 is possible via the latter; however, in some cases, they cleave Bid into tBid, which in turn stimulates mitochondrial outer membrane permeabilization (MOMP), blocking the anti-apoptotic activity of Bcl-2 and Bcl-XL, and leading to the release of cyt C, thereby activating the intrinsic pathway [107]. Oxidative stress and DNA damage, byproducts of chemo and radiotherapy, can cause tumor cells to develop drug resistance.

#### The Dual Action of Nutraceutical Compounds and Their Therapeutic Role

*In vitro* and *in vivo* studies support the idea that eating antioxidant-rich foods is a good way to reduce cancer risk. Some natural compounds have demonstrated dual action: they act as antioxidants to protect healthy cells from treatment-related oxidative stress, and as pro-oxidants in cancer cells, activating pro-apoptotic pathways. The antioxidant properties of bergamot polyphenolic fraction (BPF) are shown by its ability to reduce oxidative stress biomarkers and intracellular ROS levels [108]. Studies show that BPF can reduce MDA levels in rats fed a hyperlipidemic diet (HLD) [109] and in rats fed a high-fat diet (HFD) [110]. Bergamot’s lipid-lowering effects, alongside reductions in MDA, Lectin-like oxidized low-density lipoprotein receptor-1 (LOX-1), and protein-kinase B (PKB), indicate improved potential, especially for those on statins [111]. BPF also improves the activity of endogenous antioxidant enzymes, including GPX and SOD. By reducing peroxynitrite, it improves antioxidant defense mechanisms, thus preventing oxidative stress-induced tissue damage. High-fat Western diet-fed (WD SW) mice treated with BPF showed reduced 3-nitrotyrosine (3-NT) in their livers compared to controls; 3-NT is a biomarker of peroxynitrite oxidative damage [112]. The protective effect of BPF on dox-induced ROS accumulation in cardiomyocytes *in vivo*, leading to a reduction in Dox-induced cardiomyopathy by enhancing the resident c-kit^+^ CD45^neg^ CD31^neg^ endogenous cardiac stem cells (eCSCs) and preventing 8-hydroxy-2′-deoxyguanosine (8-OHdG) nuclear accumulation has been demonstrated [113]. Phenolic compounds found in *Cynara cardunculus* extract, including caffeic acid and chlorogenic acid are effective antioxidants [114] acting as free radical scavengers and modulating the expression of genes coding for antioxidant enzymes such as SOD, GPX, and CAT [115]. Moreover, the sesquiterpene cynaropicrin has antioxidant properties [116] and pro-oxidant action in cancer cells [117]. In fact, inhibiting thioredoxin reductase (TxR), a key enzyme in cellular redox homeostasis, causes intracellular ROS increase and triggers apoptosis in cancer cells [118]. Cynaropicrin treatment increased nuclear Nrf2 in A375 human melanoma cells, as demonstrated by *in vitro* antioxidant assays. This is linked to a time-dependent rise in the transcription of genes that code for antioxidant enzymes like glutamate-cysteine ligase (GCL) and heme oxygenase (HMOX-1) [119]. *In vivo* studies reveal the effect of cynaropicrin on the antioxidant enzymes (SOD, CAT, Glutathione Peroxidase (GSH-PX)), and on 8-OHdg in the control and cerebral ischemia–reperfusion (I/R) injury in rats, highlighting that it supports the antioxidants defense system against oxidative-stress [120]. Specifically, bergamot extract formulations with *Cynara cardunculus* show synergistic effects, potentially expanding treatment options for various diseases, including cancer, thanks to their antioxidant properties [121]. An *in vivo* study has shown the increasing level serum of GPX and SOD after 16 weeks of treatment with BPF and *Cynara cardunculus* phytocomplex, named Bergacyn^®^, accompanied by a reduction in serum MDA levels [122].

It has been shown that oleuropein is able to increase the activity of antioxidant enzymes (i.e., SOD, GPX, GRX, CAT) and non-enzymatic antioxidant systems such as GSH [123]. It is established that oleuropein’s antioxidant activity is dependent on factors such as cell type, concentration, exposure time, and oxidative stress levels [53]. In fact, its pro-oxidant action has been shown in several *in vitro* cancer cell lines, for example, MCF-7 BC cells [124], HepG2 hepatocellular carcinoma (HCC) cells [125], and HEY human epithelial ovarian cancer cell line [126], thus contributing to cell death by promoting cell damage and leading to apoptosis. Recent studies also demonstrate that oleuropein, BPF, and *Cynara cardunculus* extract lessen Dox’s cardiotoxic effects within an *in vitro* rat embryonic cardiac myoblast (H9c2) model [113,127,128]. Moreover, their contribution to upholding the integrity of cellular plasma membranes’ phospholipid bilayer could be pivotal via lipid content regulation. These natural compounds reportedly prevent endoplasmic reticulum (ER) dysfunction and promote apoptosis by modulating intracellular calcium [129]. Nevertheless, more research is necessary to verify the effectiveness and safety of these compounds for human use.

The preventative and therapeutic effects of quercetin and RV on various cancers are well-documented and dose-related [130]. At low doses, these agents exhibit antioxidant properties (ROS neutralization), but at higher doses, they stimulate ROS production, leading to apoptosis via the intrinsic pathway (cyt C release, caspase activation) and extrinsic pathway (death receptor activation) [131]. Low doses of quercetin and RV stimulate GSH synthesis and modulate antioxidant enzymes (SOD, CAT, GPX), offering protection. KEAP1 residue oxidation and subsequent inhibition leads to Nrf2 accumulation in the cytoplasm, followed by nuclear translocation and ARE activation [132]. Through AMPK and silent mating type information regulation 2 homolog 1 (SIRT1) activation, RV inhibits the mammalian target of rapamycin (mTOR) and deacetylates/activates forehead transcription factor O subfamily member 3a (FOXO3a), boosting antioxidant enzyme gene expression [133]. While at high doses, they interfere with mitochondrial function, generating dissipation of membrane potential, with the release of cyt C [134]. By suppressing intracellular ROS accumulation, RV protected H9c2 cardiac cells form Dox-caused toxicity [135]. Because of its strong antioxidant properties, quercetin enhances Dox’s antitumor activity by inhibiting topoisomerase II (Topo II) and intercalating with DNA [136].

Several studies highlighted the biphasic effect of ferutinin [137]: low doses, in fact, show antioxidant and protective effects on healthy cells, decreasing oxidative damage. Ferutinin pretreatment at a low dose significantly reduced Dox-induced cardiotoxicity in H9c2 cells, according to Macrì et al. [138]. In addition, ferutinin at low concentrations demonstrated hyperproliferative effects with phytoestrogenic action in MCF-7 BC cells. At higher concentrations, ferutinin can increase oxidative stress in cancer cells with antiproliferative effect [139], showing a lower toxicity in healthy cells with respect to cancer cells. Bax upregulation and cyt C release, hallmarks of mitochondrial apoptosis, are dose-dependently increased by ferutinin [140]. It shows selective toxicity toward cancer cells over healthy cells, with effects varying by dose and cell type. Specifically, high ferutinin levels suppressed the growth of MCF-7 (estrogen-dependent) and MDA-MB-231 (estrogen-independent) BC cells [141]. Furthermore, ferutinin pretreatment decreased ROS and MDA buildup in lipopolysaccharide-stimulated (LPS) neurons and shielded human oligodendrocytes (MO3.13) and neurons (SH-SY5Y) from LPS-caused oxidative stress [142]. Therefore, further *in vitro* and *in vivo* research is needed to clarify the dose-dependent mechanisms of nutraceuticals’ dual effects on cancer cells (Table 2) and their potential as cancer therapy adjuvants.

## 3. Highlights of the Inflammatory Process

Inflammation plays a crucial role in cancer development and metastasis, thus a key focus for cancer therapies. The growth of blood vessels, stimulated by cytokine production, aids carcinogenesis, while the resulting ROS leads to DNA damage. Moreover, the promotion of resistance mechanisms in cancer cells by inflammation can lead to chemoresistance.

This occurs both through increased DNA damage due to inflammation, with activation of oncogenic bypass signaling pathways (e.g., mesenchymal–epithelial transition factor (C-MET), PI3K/AKT, MAPK), and through the action of some mediators of inflammation, including IL-6 and interleukin-1 alpha and interleukin-1 beta (IL-1α/β), which modulate apoptotic and autophagic processes [144]. Tissue damage triggers inflammation—the body’s complex response involving a cascade of pro- and anti-inflammatory mediators (e.g., cytokines, prostaglandins) to various insults (ischemia, trauma, infection, toxins).

The inflammatory process recruits microcirculation ECs and white blood cells, a process driven by molecular signals and balanced levels of pro- and anti-inflammatory mediators which are essential to maintain homeostasis [145]. A persistent inflammatory trigger or a failed initial response can shift an acute inflammatory to a chronic process. Aggressive cancer phenotypes, marked by increased survival, proliferation, invasion, angiogenesis, and metastasis, are often facilitated by the pro-cancer microenvironment generated from chronic inflammation.

The inflammatory response, characterized by toll-like receptor (TLR) activation, is linked to and modified by dysbiosis [146], NOD-like receptors (NLRs), C-type lectin receptors (CLRs), and retinoic acid-inducible gene (RIG)-I-like receptors (RLRs) by neutrophils/macrophages in response to pathogen-associated molecular patterns (PAMPs) [147].

Leukocyte recruitment and infiltration are mediated by chemokines like interleukin-8 (IL-8) and macrophage-inflammatory protein 2 (MIP-2) alongside inflammatory mediators including complement C5a complement fragments, platelet-activating factor (PAF), and leukotriene B4 (LTB4) [148] from the venous system to sites of tissue damage, and there is their attachment to the ECM. Activated ECs express adhesion molecules, such as the intercellular adhesion molecule 1 (ICAM-1) and intercellular adhesion molecule 2 (ICAM-2), facilitating leukocyte adhesion and diapedesis. This process involves adhesion molecules (i.e., L-P- and E-selectin) and chemokines/cytokines (i.e., TNF-α, TGF-β, IL-1β, IL-6) secreted by activated macrophages, which regulate inflammatory cascade. The pro-inflammatory phenotypes in ECs and fibroblasts are mainly triggered by TNF-α, IL-6, and IL-1β, which activate TNFR, interleukin-6 receptor (IL-6R), and interleukin-1 receptor (IL-1R), resulting in the activation of NF-κB, MAPK, and Janus kinase/signal transducer and activator of transcription (JAK-STAT) signaling pathways [149].

### 3.1. Hallmarks of Cancer Inflammation

The research focuses on understanding the mechanisms through which inflammation impacts cancer, aiming to identify more selective therapeutic targets within specific pathways [144]. Findings from basic and clinical research demonstrate the importance of inflammatory molecules in the initiation and advancement of multiple cancer types, including breast, lung, and liver cancers [150]: these molecules could impact every phase of tumor growth, from malignant change to metastasis [151]. Conversely, acute inflammation may inhibit cancer progression: indeed, it can stimulate the immune system to help remove tumor cells. The onset of chronic inflammation increases the risk of developing highly invasive cancers. In particular, an inverse correlation between MedDiet and chronic inflammation, with lower levels of inflammatory biomarkers (i.e., CRP and IL-6) and lower risk of several cancers, has been reported [152]. Inflammation can promote tumor initiation, due to infections (e.g., Human Papillomavirus (HPV), *Helicobacter pylori* (*H*. *pylori*), Hepatitis B Virus (HBV), Hepatitis C virus (HCV) or carcinogens (e.g., cigarette smoke, toxic substances, ionizing radiation) [153]. Conditions such as diabetes, hyperlipidemia, and CVD are associated with low-grade inflammation that may predispose to the risk of developing cancer (e.g., BC, HCC, pancreatic cancer (PC)) [154]. The activation of Signal Transducer and Activator of Transcription proteins (STAT), especially Signal Transducer and Activator of Transcription 3 (STAT3), a transcription factor regulating genes related to cell growth, survival, and immunity, is heavily involved in cancer development across many tissues and inflammatory responses in cancers of the stomach, liver, lung, colon, and pancreas. ROS and RNS, substances produced by leukocytes and other phagocytic cells, can cause irreversible DNA damage (oncogenic mutations like RAS and myc, and the inactivation of p53 and retinoblastoma protein 1 (RB1)), increasing cancer risk. The cytokine MIF, released from macrophages, T lymphocytes, and tumor cells, can aggravate DNA damage and the accumulation of oncogenic mutations [155]. By suppressing p53’s transcriptional activity, MIF interacts with it and promotes the survival of damaged cells. By suppressing pro-apoptotic gene activation and stimulating cell proliferation through PI3K/AKT and NF-κB signaling, this mechanism contributes to tumor development. The release of pro-inflammatory cytokines (TNF-α, IL-1, IL-6, IL-8, and interleukin-23 (IL-23)) from macrophages and T-lymphocytes indirectly leads to genetic instability via epithelial cell ROS production [151]. NF-κB signaling is activated via TNFR and IL-1 receptor engagement by TNF-α and IL-1 cytokines, thereby promoting a pro-inflammatory phenotype in ECs and fibroblasts. The JAK/STAT3 pathway is mainly activated by IL-6 and IL-23, contributing to the development of inflammatory tumors. In addition, the binding of IL-22 to its receptor (Interleukin-22 Receptor Subunit 1(IL-22R1)/Interleukin-10 Receptor Subunit 2 (IL-10R2)) triggers the janus kinase 1 and tyrosine kinase 2 (JAK1/TYK2) pathway, causing STAT3 phosphorylation and subsequent nuclear translocation to activate target genes involved in epithelial cell survival and regeneration. The roles of NF-κB and STAT3 pathways in tumor formation are widespread across many tissue types and strongly associated with inflammation in cancers of the stomach, colon, liver, lung, and pancreas, influencing cell proliferation, survival, angiogenesis, and immune evasion [156]. The TME comprises cancer cells, stroma, blood vessels, CAFs, ECs, pericytes, dendritic cells (DCs), and activated macrophages; these components are crucial for both antigen-specific immunity and immune tolerance, bridging innate and adaptive immunity. The TME significantly involves TAMs, which are derived from circulating monocytic precursors and are directed toward the tumor and are induced by interferon-gamma (IFN-γ)-mediated classical activation or alternatively by the helper T 2 cells (Th2) and cytokines like interleukin-4 (IL-4) and interleukin-13 (IL-13). TAMs can stimulate tumor cell proliferation, promote angiogenesis, and promote invasion and metastasis. Inflammatory-TME also includes polymorphonuclear leukocytes, and various inflammatory cells such as T lymphocytes (sometimes B cells) [157,158]. Consequently, the link between oxidative stress, chronic inflammation, and cancer development is clear. Recent findings indicate a link between the aberrant activation of Wntβ/catenin signaling and the progression of tumors and chronic inflammatory diseases. Research into Wnt/β-catenin signaling reveals opposing roles for peroxisome proliferator-activated receptor-gamma (PPARγ), which is downregulated in chronic inflammation and cancer [159,160]. PPARγ’s role is context and tissue-specific, with evidence suggesting it can function as an oncosuppressor in certain situations, causing cell cycle arrest and apoptosis while lowering cell invasion, migration, and inflammation [161]. Deregulation in pro-inflammatory or hyperlipidic conditions promotes oncogenesis [162]. Conversely, the NF-κB pathway upregulates pro-inflammatory cytokines (interleukin-6 (IL-6), VEGF, (IL-8)), Inducible nitric oxide synthase (iNOs), TGF-β, indirectly boosts WNT/β-catenin, thus fueling inflammation and tumor growth. Survival and prognosis may be indicated by the grade of tumor infiltration, which varies by tumor type.

### 3.2. Modulation of Inflammatory Pathways by Nutraceutical Compounds from the Mediterranean Diet

**Figure 2:**
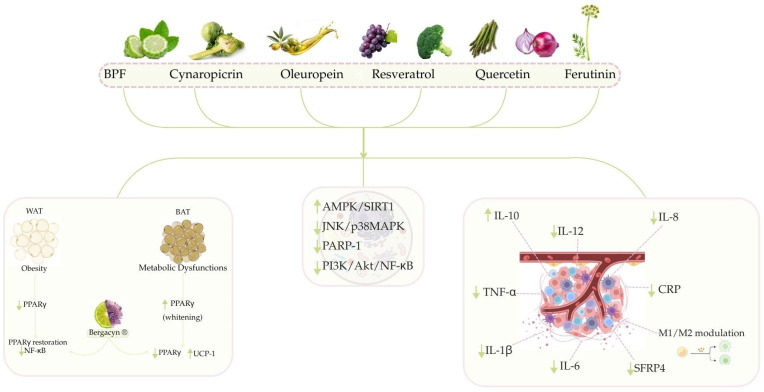
BPF, cyn, oleuropein, RV, quercetin, and ferutinin are able to modulate inflammatory pathways including AMPK/SIRT1, JNK/p38MAPK, PARP-1, and PI3K/Akt/NF-κB; modulate the secretome of tumor cells by reducing the secretion of pro-inflammatory cytokines (IL-12, IL-8, CRP, SFRP4, IL-6, IL-1β, TNF- α) and increasing the secretion of anti-inflammatory cytokines (IL-10), and modulate the activation state of macrophages. Bergacyn^®^ restored PPARγ levels and prevented NF-κB overexpression in obese WAT, where PPARγ is significantly reduced. Whereas, in BAT, despite the increased expression of PPARγ, during metabolic dysfunction and “whitening,” Bergacyn^®^ reduced PPARγ. Restoration of adipose tissue homeostasis and regulation of PPARγ by Bergacyn^®^ could reduce inflammation and metabolic signals involved in some cancers. The arrows indicate decrease (↓) and increase (↑), respectively. Bergamot polyphenolic fraction (BPF); cynaropicrin (cyn); resveratrol (RV); AMP-activated protein kinase/silent mating type information regulation 2 homolog 1 (AMPK/SIRT1); c-Jun amino-terminal kinase/p38 mitogen-activated protein kinase (JNK/p38MAPK); poly (ADP-ribose) polymerase-1(PARP-1); phosphatidylinositol 3-kinase/protein kinase B/Nuclear factor kappa-light-chain-enhancer of activated B cells (PI3K/Akt/NF-κB); interleukin-12, -8, -6, -1β, -10 (IL-12, -8, -6, -1β, -10); C reactive protein (CRP); secreted frizzled-related protein 4 (SFRP4); tumor necrosis factor alpha (TNF- α); peroxisome proliferator-activated receptor-gamma (PPARγ); brown adipose tissue (BAT); white adipose tissue (WAT).

The effect of diet on acute and chronic inflammation is associated with the development of cancer. [163]. The anti-inflammatory properties of numerous natural compounds suggest their potential use as supplemental components within alternative anti-inflammatory treatment systems (Figure 2).

#### 3.2.1. Anti-Inflammatory Activity of Citrus Bergamia

*Citrus bergamia* derivatives have demonstrated anti-inflammatory activity, linked to AMPK/SIRT1 axis activation [164]. The activation of AMPK, a key regulator of energy and senescence, is linked to changes in inflammatory factors such as IL-6 and TNF-α [133]. Importantly, BPF might reduce levels of serum glucose, transaminases, gamma-glutamyl transferase (GGT), and inflammatory biomarkers such as TNF-α and CRP [165]: this prevents worsening liver inflammation and fibrosis [112]. Reduced hepatic inflammation, via IL-6 reduction and Interleukin-10 (IL-10) mRNA upregulation, has been linked to BPF supplementation [166]. By lowering phospho-JNK and phospho-p38 MAPK, it can control the release of pro-inflammatory cytokines such as IL-1β. The reduction in inflammation and hepatocellular ballooning, key to cancer initiation, also supports this effect [112,150]. This effect may also be due to BPF’s antioxidant activity, which lowers excessive poly (ADP-ribose) polymerase-1 (PARP-1) activity. In disease states, the second choice has been observed to decrease the levels of the MAPK inhibitor mitogen-activated protein kinase phosphatase 1 (MKP-1), leading to elevated inflammation. Consequently, BPF’s anti-inflammatory effect stems from the decreased PARP-1 repression of MKP-1, leading to JNK/p38 MAPK inhibition [167]. Findings from a randomized, double-blind, placebo-controlled study in type 2 diabetes mellitus/non-alcoholic fatty liver disease (T2DM/NAFLD) patients show that Bergacyn^®^ improves NAFLD and liver fibrosis markers by modulating inflammatory biomarkers [122].

#### 3.2.2. The Beneficial Effects of Cynaropicrin and Bergacyn^®^ to Counteract Inflammation

Recently, *in vivo* studies evaluated Bergacyn^®^’s potential for tissue-specific PPARγ modulation. In obese white adipose tissue (WAT), where PPARγ is significantly reduced, Bergacyn^®^ restored PPARγ levels and prevented NF-κB overexpression. Despite increased PPARγ expression in brown adipose tissue (BAT) during metabolic dysfunction and “whitening,” Bergacyn^®^ downregulates PPARγ, thus promoting a thermogenic and metabolically active state [121]. PPARγ’s involvement in colorectal, lung, breast, prostate, and pancreatic cancer (proliferation, differentiation, and inflammation) makes the tissue-specific Bergacyn^®^ modulation data highly impactful [168,169]. Moreover, the inflammatory microenvironment, a consequence of obesity-linked adipose tissue dysfunction, can promote tumor progression. Therefore, the restoration of adipose tissue homeostasis and PPARγ regulation by Bergacyn^®^ could lessen inflammation and metabolic signals involved in certain cancers [121] (Figure 2). Several studies highlight cynaropicrin’s involvement in the inhibition of the NF-κB transcriptional activation pathway [170]. *In vitro* studies on the murine macrophage cell line RAW264.7 show its ability to inhibit inflammatory cytokine secretion following the induction of inflammation with LPS [171]. In the A375 cell line treated with cynaropicrin, the p65/RelA protein, a member of the NF-κB family, was reduced [119]. *In vivo* studies report that dosage-based cynaropicrin treatment on rats with cerebral I/R injury-induced significantly reversed the increased level of the pro-inflammatory cytokines (TNF-α, IL-6, and IL-1β), and appreciably reduced the mRNA level of NF-κB [120].

#### 3.2.3. Anti-Inflammatory Activity of Oleuropein

*In vitro* oleuropein has been shown to reduce the secretion of pro-inflammatory cytokines and inflammatory mediators [172,173]. In a RAW264.7 macrophage line activated with LPS (M1 phenotype), oleuropein treatment reduced Interleukin-12 (IL-12) and TNF-α levels in the cell culture supernatant and the oleuropein action on readjusting the M1/M2 macrophage polarization towards the anti-inflammatory M2 phenotype has been reported [174]. RAW264.7 cells were used to assess oleuropein’s ability to reduce LPS-induced inflammation by reducing levels of IL-1β, IL-6, and TNF-α [175,176]. Oleuropein pretreatment in LPS-activated Human Umbilical Vein Endothelial Cells (HUVECs) has been shown to potentially lower mRNA levels of pro-inflammatory cytokines like IL-1β, IL-6, TNF-α, and IL-8 [172]. Several *in vivo* studies demonstrated oleuropein’s anti-inflammatory action via reduced pro-inflammatory cytokine levels [177,178]. In addition, oleuropein treatment effectively mitigated the adverse effects of inflammation in obese mice. Specifically, the treatment led to higher PPARγ levels in fat and the liver, and lower JNK-1 levels in the liver [179].

#### 3.2.4. The Beneficial Role of Resveratrol and Quercetin to Counteract Inflammation

Recent research indicates RV and quercetin, at varying doses, decrease inflammation by impacting SFRP4, a protein involved in obesity, diabetes, and pancreatic inflammation, thus highlighting a connection between obesity-driven inflammation and dysfunctional insulin response. Resveratrol and quercetin’s potent antioxidant and anti-inflammatory effects suggest their potential as pancreatic oxidative stress drug candidates [180].

Extensive research supports the growing potential of natural compounds like resveratrol and quercetin to treat inflammation; these compounds reduced weight gain, dyslipidemia, and inflammation in a study of obese rats. Reduced body weight gain, adipocyte size, and adipose tissue mass, as well as improved serum dyslipidemia, are linked to the anti-obesity effects of quercetin and resveratrol. A quercetin and resveratrol blend’s obesity-reducing effects are linked to its anti-inflammatory properties; it decreases adipokine secretion, activates AMPKα1/SIRT1 signaling, and thus may reduce HFD-induced obesity and inflammation [181].

Further evidence supports quercetin’s neuroprotective effects, potentially by promoting microglia/macrophage M2 polarization via the PI3K/Akt/NF-κB pathway. These results suggest quercetin may be a useful treatment for ischemic stroke [182].

Cardiac hypertrophy in mice was induced by implanting Ang II osmotic pumps. Researchers investigated primary neonatal rat cardiomyocytes and heart tissues to understand resveratrol’s mechanism in preventing Ang II-induced cardiac hypertrophy. This research reveals important new mechanisms by which resveratrol protects against Ang II-induced cardiac hypertrophy, specifically by blocking NF-κB signaling and pro-inflammatory cytokines. The findings add to the evidence that REV may be a promising drug for treating cardiac hypertrophy and heart failure [183].

#### 3.2.5. Preliminary Evidence of Anti-Inflammatory Activity of Ferutinin

Antioxidant, anti-inflammatory, and anticancer effects of bioactive compounds from plants and herbs were demonstrated *in vitro* and *in vivo*.

Reducing oxidative stress lowers the risk of developing various diseases, including cancer and chronic inflammatory diseases. The antioxidant activity of flavonoids, phenolic acids, stilbenes, tannins, and lignans (phenolic plants) is demonstrated *in vivo* and *in vitro*: the presence of hydroxyl groups on their aromatic rings is responsible for reducing oxidative stress, with antioxidant capacity increasing with the number of these groups. Specifically, bergamot flavonoids have demonstrated pleiotropic effects, reducing oxidative stress and inflammation at the cellular and tissue levels. Moreover, *in vitro* data suggests antioxidant activity in monoterpenes, sesquiterpenes, and diterpenes, bioactive compounds extracted from various plants and fruits. In fact, sesquiterpenes show significant anticancer, anti-inflammatory, and bactericidal effects, suggesting an important role in human diseases. Different experiments suggest that sesquiterpenes offer protection in low doses, while high doses cause severe toxicity; the bipolar effect is a dose-dependent increase in the permeability of lipid bilayers and mitochondrial membranes to cations—particularly divalent cations like calcium—caused by sesquiterpenes, which are important in various pathophysiological processes [137]. Furthermore, ferutinin demonstrates anti-inflammatory effects by reducing oxidative stress. The anti-inflammatory effects of ferutinin were evaluated using an edema model. Specifically, the reduction in inflammation during the early stages was significant, likely due to histamine and serotonin. Notably, at this stage, ferutinin demonstrated a significantly more potent anti-inflammatory effect compared to the aspirin control dose. This study implies ferutinin’s anti-inflammatory action likely results from blocking histamine and/or serotonin activity [184]; however, further studies are needed to better explain the pathway under these mechanisms.

## 4. Cell Cycle Regulation: Key Mechanisms and Links to Tumorigenesis

A misregulation in cell cycle regulation can promote the development of diseases, including cancer. The cell cycle is controlled by molecular mechanisms involving distinct CDK-cyclin complexes, arising from the interplay between cyclin-dependent kinases (CDKs) and cyclins [185], and through three checkpoints: (i) G1 checkpoint, (ii) G2 checkpoint, and (iii) M checkpoint, also known as spindle assembly checkpoint (SAC). The G1 checkpoint’s activity is determined by growth factors, capable of both promoting and suppressing cell growth, thus monitoring the transition between the G1 phase and the S phase. The regulation of the G1 checkpoint hinges on the crucial roles played by CDKs and tumor suppressor proteins, notably p53 and pRb. If a cell identifies damage or irregularities, it activates DNA repair; if this fails, apoptosis is triggered.

The tumor suppressor p53 controls cell metabolism, redox balance, and DNA repair, inducing cell cycle arrest and cell death. Cell cycle arrest results from p53 binding to upstream promoter regions of the p21 gene, leading to its transcriptional activation; in particular, p21 protein inhibits cyclin E/Cdk2 and cyclin D/Cdk4/6 complexes, resulting in cell cycle arrest in the G1 phase [186]. Findings suggest that zinc finger protein 57 (ZFP57), a key regulator of BRCA1 expression in ovarian cancer, might impact the G1 cell cycle checkpoint [187]. The silencing of CEP192, a novel gene involved in the development of NAFLD and HCC, has been recently shown to cause cell cycle arrest in the G0/G1 phase of cancer cells, thereby decreasing their proliferation and self-renewal. These results reveal the onco-immunological involvement of CEP192 in the establishment of a TME [188]. The cell uses the G2 checkpoint to confirm successful DNA replication before beginning mitosis. Its regulation involves a complex network including CDKs and checkpoint kinases (Chk1 and Chk2), along with other proteins and signaling pathways. Overexpression of polo-like kinase 1 (PLK1), a crucial mitotic checkpoint protein for cell cycle progression, is associated with unfavorable prognoses in some cancers (e.g., pancreatic adenocarcinoma) [189]. The M checkpoint monitors kinetochore–microtubule binding during mitosis; its regulation involves Mad, Bub proteins, CDKs, and other signaling pathways [190]. The regulation of all cell cycle phases, including G1/S and G2/M transitions, by the PTEN oncosuppressor gene is crucial for cell proliferation and survival. Furthermore, it plays a role in preserving chromosome structure, safeguarding the genome against structural and numerical chromosomal instability (CIN). CDKs, specifically A, B, D, and E, are key to the molecular mechanisms governing cell cycle control. Cyclins, each produced and degraded at specific cell cycle stages, bind to particular CDKs, forming active complexes essential for each phase and their activity is positively modulated by cyclins and inhibited by CDK inhibitors (CKIs). Transcriptional and post-translational modifications, along with the ubiquitin-proteasome system’s rapid degradation of cyclins and CKIs, control the activation state. Dysregulation of cyclin expression and activity, and mutations or changes in the genes that regulate checkpoints, can lead to uncontrolled cell division and contribute to cancer development [191].

### 4.1. Mechanistic Insights into Cell Cycle Alterations in Cancer Development

Cell cycle dysregulation arises from multiple pathways: checkpoint failures, spindle checkpoint defects, CDK/cyclin mutations, abnormal cyclin D and E levels, and telomerase-driven immortality. The initial step in tumor formation is cell immortalization, or the evasion of senescence; the immortalization is related to the activity of enzyme telomerase, which increases telomere length at the ends of chromosomes [192]. Common alterations in the cell cycle include elevated levels of CDK4/6 and CDK2, both vital kinases in the G1 to S phase progression. Phosphorylated Rb underlies these alterations, leading to E2F release and activation of genes essential for cell cycle’s S phase initiation. Overactive complexes correlate with boosted cancer cell growth, aging, migration, and blood vessel formation, alongside apoptosis resistance and therapy resistance (like against CDK inhibitors) in cancers such as breast and ovarian cancers [193]. Genes regulating cell cycle checkpoints, including p53 and FRP1, also show mutations or deletions in cancer cells. The lack of p53 crucially affects cell cycle checkpoint regulators, leading to the increased expression of genes such as p21 (a growth inhibitor) and pro-apoptotic genes, mainly after DNA damage halts the cell cycle at G1 [186]. Uncontrolled cell cycle progression may result from the loss of oncosuppressor genes, including Rb1, p16INK4A, p15INK4B, and p14ARF. Furthermore, mutations in p16, Rb loss, or increased CDK2 activity can lessen the effectiveness of CDK4/6 inhibitors (e.g., palbociclib, ribociclib, abemaciclib). This has prompted the development of combination, or second-generation, strategies to overcome therapeutic resistance [193]. The link between checkpoint failure and cancerous phenotypes in normal cells is established; however, the specific genetic alterations responsible are still unknown. Furthermore, the direct causal link between checkpoint malfunction and cell transformation, versus the involvement of other regulatory genes after immortalization, requires further investigation. In cancer treatment, genes involved in checkpoints may be useful as diagnostic tools and as targets for new drugs [194].

### 4.2. Nutraceutical-Induced Cell Cycle Modulation in Cancer and Chemotherapy—Induced Cytotoxicity

**Figure 3:**
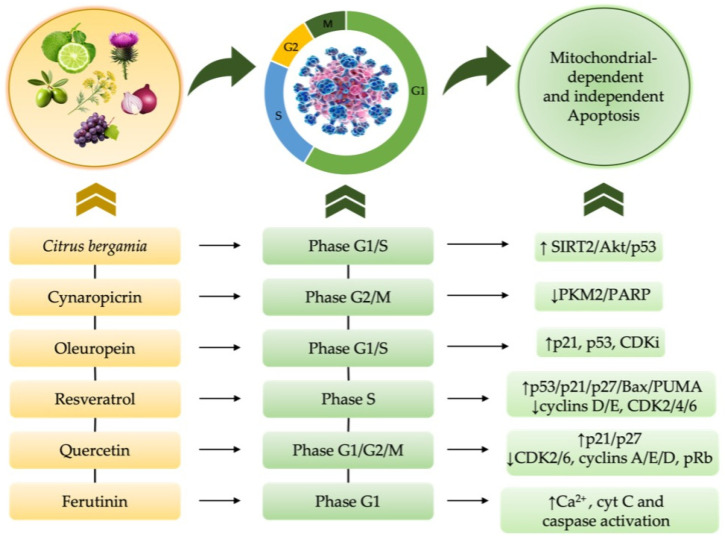
The anti-proliferative effects of natural extract derivatives on different cancer cell types were evaluated through cell cycle arrest in multiple *in vitro* studies. *Citrus bergamia* has been shown to possess an antiproliferative effect by arresting the cell cycle in the G1/S phase through the modulation of the SIRT2/Akt/p53 pathway. Cynaropicrin arrested the cell cycle in the G2/M phase by modulating the PKM2/PARP pathway. Oleuropein exerts an antiproliferative effect by arresting the cell cycle in the G1/S phase by increasing p21/p53/CDKi. RV treatment arrested the cell cycle in the S phase by modulating p53/p21/p27/Bax/PUMA and the reduction in cyclins D/E and CDK 2/4/6. Treatment with quercetin causes cell cycle arrest in G1/G2/M phases with the modulation of p21/p27 and reduction in CDK 2/6 and cyclins A/E/D and phosphorylation of Rb. Ferutinin treatment has been shown to exsert an antiproliferative effect leading to cell cycle arrest in the G1 phase by increasing intracytoplasmic Ca 2+ concentration, inducing cyt C release and caspases activation. The arrows indicate decrease (↓) and increase (↑), respectively. Silent mating type information regulation 2 homolog 2 (SIRT2); protein kinase B (Akt); tumor protein p53 (p53); Cynaropicrin (Cyn); Pyruvate kinase M2 (PKM2); poly-ADP-ribose-polymerase (PARP); tumor protein p21/p53; Cyclin-dependent kinase inhibitor (CDKi); Resveratrol (RV); p53/p21/p27; BCL2 associated X, apoptosis regulator (Bax); p53 upregulated modulator of apoptosis (PUMA); Cyclin-dependent kinases (CDK 2/4/6); Cytochrome C (Cyt C).

The effects of natural compounds on inhibiting tumor cell proliferation by modulating specific cell cycle checkpoints and interfering with mitotic progression have been shown; their effects on cell cycle regulation are reported in Table 3. This suggests both their potential use as coadjuvants in the treatment of some types of cancer, and as a treatment to reduce the toxic effects induced by chemotherapeutic drugs in non-cancerous cells [195] (Figure 3).

The anti-proliferative effects of *Citrus bergamia* natural derivatives on different cancer cell types were evaluated through cell cycle arrest in multiple *in vitro* studies [143]. Human leukemia THP-1 cells treated with Flavonoid-Rich Extract (FRE) of BJ showed cell cycle arrest in the S phase, leading to apoptosis through the SIRT2/AKT/p53 pathway [196]; in addition, *Citrus bergamia* juice decreased human neuroblastoma SH-SY5Y cell growth *in vitro* via G1 cell cycle arrest and reduced cell adhesion [197]. Animal studies showed that FRE of BJ decreased levels of baculoviral inhibitor of apoptosis repeat-containing 5 (Birc5) and the cell cycle regulator p21, potentially increasing tumor cell death [198].

Other evidence highlighted that cynaropicrin could inhibit cellular growth by cell cycle arrest, mainly in the G2/M phase, in thyroid cancer cell lines CAL-62, 8505C, and SW1736 cells, by a dose- and time-dependent way [199]. This also demonstrates PKM2 inhibition in A549 cells, causing increased p53, decreased DNA repair enzyme PARP, with a subsequent cell cycle arrest [200].

Oleuropein significantly modulates the cell cycle, notably by upregulating CDK inhibitor levels, thus causing cell cycle arrest, and by increasing p53 and p21 levels, which then affects gene expression to activate both intrinsic and extrinsic apoptosis pathways [201]. *In vitro* investigations using MCF-7 and MDA-MB-231 BC cells revealed that oleuropein treatment induced cell cycle arrest at the G1 and S phases [202]; its effects on MDA-MB-231 and MDA-MB-468 BC cell lines include cell proliferation inhibition, apoptosis induction through S-phase arrest, and caspase-1, -4, and -14 expression [203].

Several *in vitro* studies [209] indicate quercetin’s role in modulating the cell cycle. Myeloid cell line p39 and osteosarcoma cells HOS showed increased G1 cell cycle arrest after treatment, accompanied by decreased CDK-2, -6, cyclins A, D, E, Rb phosphorylation, and increased p21 and p27 expression [210]. Furthermore, quercetin triggered G1 cell cycle arrest in oral squamous cell carcinoma cell lines YD10B and YD38 OSCC [211].

It has been suggested that quercetin treatment leads to G2/M cell cycle arrest in T47D and KON BC cells and A375 melanoma cells [212].

Moreover, RV’s *in vitro* effects included enhanced G1 to S phase transition, S phase cell cycle arrest, and reduced cancer cell proliferation and viability. Prostate cancer cells (LNCaP and PC-3) treated with RV showed reduced expression of cyclins D1 and E, CDK4, and cyclin D1/CDK4 kinase activity [204]. RV’s ability to decrease A431 and colon cancer cell proliferation has been linked to lower levels of cyclins D1/D2/E2, CDK2/4/6, and higher levels of p21 and p27 [205]. RV was found to activate p53 along with its target genes p21, p27, Bax, PUMA, MDM2, and cyclin G [206]. Therefore, RV may stimulate the G1-to-S phase transition in BC cells, subsequently halting their progression through the S phase, thus diminishing their proliferation and viability [207]. In addition, it has been reported that RV reduces *Versican* (VCAN) secretion by CAFs, presumably by modulating the cell cycle [208].

**Figure 4:**
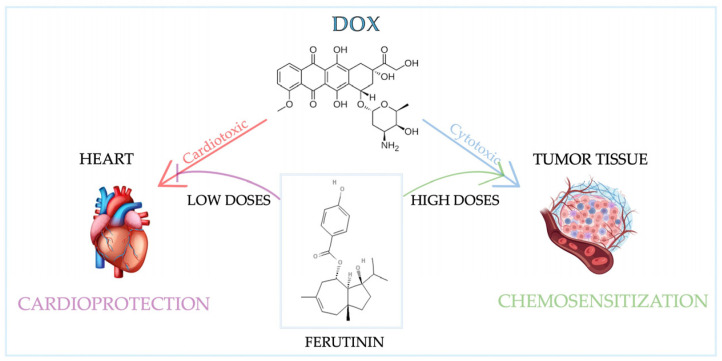
The tissue-dependent and dose-dependent effect of ferutinin. At low doses, ferutinin ca counteract the cardiotoxic effect of Dox. At high doses, ferutinin can enhance Dox chemosensitization in tumor tissues. Doxorubicin (Dox).

Exposure to ferutinin leads to a decreased G1 phase and increased cell death, modifying the cell cycle profile [140]. In addition, it was shown that ferutinin, thanks to its cell-type dependent activity, restores the cell cycle in H9c2 myoblasts incubated with Dox [138], suggesting its protective role in cell cycle progression, highlighting its potential as a therapeutic agent in cancer treatment and in mitigating chemotherapy-induced cardiac damage (CIC) (Figure 4) [213].

## 5. The Human Microbial Landscape in Cancer Development

The human microbiota comprises approximately 300 bacterial, fungal, and viral species, totaling almost 40 trillion microorganisms [214,215]. Over 97% of the human microbiota is located in the gastrointestinal tract, mainly the colon, and is known as the gut microbiota. In addition, current findings demonstrate a diverse microbiome within previously considered sterile tumor tissues (lung, breast, liver), which is closely associated with oncogenesis [216,217]. Consequently, the intratumoral microbiota (ITM) concept emerged and ITM’s anti- and pro-tumorigenic effects have been demonstrated through mechanistic studies [218]. Tumor-inhibiting metabolites are produced by T and NK cells activated by the ITM. These may raise oxidative stress, causing genomic instability and mutations, affecting epigenetics, weakening immunity, and stimulating inflammation, thus, leading to tumor initiation and progression [219], representing an integral part of TME [218]. Tumors in mucosal organs, including the colon, cervix, esophagus, and lung, are colonized by the ITM [220].

Non-mucosal organs such as the pancreas, liver, breast, and prostate also contained ITM, which suggests the existence of both adjacent tissue invasion and hematogenous spread during colonization [218]. This supports the hypothesis that TME colonization by the ITM may result from malformed blood vessels [217]. Tumor subtype differences in intratumoral microbial composition have been demonstrated [221]. Tumors can arise from viral infections and persistent bacterial inflammation, such as that caused by *H*. *pylori* and gastritis.

Evidence on the colon cancer microbiota has shown the presence of bacteria *Bacteroides fragilis*, *Escherichia coli* (*E*. *coli*), and *Fusobacterium nucleatum*—frequently detected in malignant pancreatic and BC—and fungal species such as *Candida albicans* [219]. The species of *Blastomyces* are prevalent in lung cancers, while the species of *Malassezia* are abundant within BC [216]. TME regulation originates from both the tumor and gut microbiota, impacting immune responses and cancer cell metabolism; consequently, this crosstalk between intratumoral and gut microbiota might elicit specific immune reactions [222]. Metagenomics, using next-generation sequencing (NGS) and computational analysis of 16S rRNA amplicons, has enabled the characterization of gut microbiota diversity and abundance [223,224]. *Firmicutes* and *Bacteroidetes* are the most prevalent phyla among the roughly 1000 bacterial species in the gut microbiota. The gut’s microbiome plays a critical role in numerous physiological processes, including vitamin production, the metabolism of food components, protection from pathogens, and the shaping of the host’s immune system [225]. The pharmacomicrobiomic concept has established the gut microbiota’s role in influencing drug effects, such as chemotherapy. In fact, the gut’s microbes can alter drug metabolism, impacting their absorption, effectiveness, or safety [226]. Dysbiosis, an imbalance of gut microbiota, is linked to cardiovascular, metabolic, and neurodegenerative diseases, as well as cancer [227]. Studies of the gut microbiome via metabolomics and metagenomics show its dual impact on cancer prevention, onset, and therapy, having both suppressing and oncogenic activity [228]. Several studies indicate that communication between colorectal cells and gut bacteria is vital for the body’s overall health and immune response, especially in metabolism. The influence of gut microbiota on cancer initiation, progression, and metastasis, as well as its potential in cancer treatment, is a promising area in precision medicine [229].

### Emerging Connections and Therapeutic Perspectives of Gut Microbiota and Nutraceuticals in Cancer

**Figure 5:**
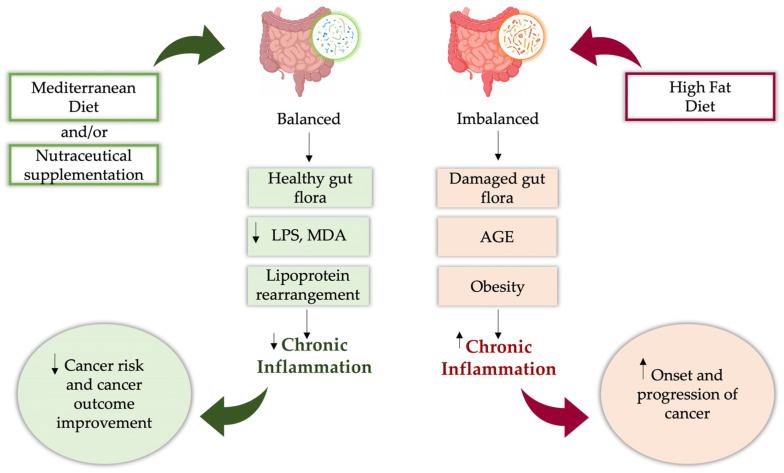
Mediterranean-style dietary model and/or nutraceutical supplementation are directly related to health gut microbiota. The MedDiet, rich in fiber and natural compounds such as polyphenols, supports a balanced microbiota, reduces inflammation, LPS and MDA levels, promotes lipoprotein rearrangement, and contributes to reduction in cancer risk and cancer outcome improvement. Controversially an HFD alters the composition of the microbiota, promoting AGEs production, with increased chronic inflammatory state—associated with obesity, and activating the gut–bone marrow–tumor axis, resulting in increased onset and progression of cancer. The arrows indicate decrease (↓) and increase (↑), respectively. Mediterranean Diet (MedDiet); lipopolysaccharide (LPS); malondialdehyde (MDA); high fat diet (HFD); advanced glycation end product (AGE).

An interplay between the gut microbiota, diet-induced inflammation, and cancer pathogenesis has been found. In fact, an altered microbiota associated with chronic inflammation in colon, pancreatic, breast, gastric, liver, and prostate cancer, has been identified. Consequently, investigations on nutraceutical compounds potentially able to modulate the microbiota, with implications on cancer pathogenesis, are crucial. A correlation between diet and healthy gut microbiota has been demonstrated, highlighting the implication of dietary natural compounds in affecting cancer through the gut microbiota [230]; thus, this relationship between diet and cancer through the gut microbiota may provide new insights for cancer treatment [231] (Figure 5). Specifically, metabolic changes like obesity are linked to persistent low-level inflammation, a connection between obesity and cancer supported by many studies. The increasing prevalence of excessive fat dietary intake is linked to the adoption of HFD in many countries, with a lack of fiber intake [232]. In comparison to traditional rural populations, HFD populations showed reduced bacterial diversity [233]. HFDs impact gut microbiota to produce more leucine, subsequently activating the mammalian target of rapamycin complex 1 (mTORC1) in bone marrow, leading to increased polymorphonuclear myeloid-derived suppressor cells (PMN-MDSC) differentiation and faster BC growth through the gut–bone marrow–tumor axis.

Evidence indicates a positive correlation between good adherence to the MedDiet and the health of the gut microbiota and could potentially represent an important pattern in reducing the incidence of a range of diseases, including cancer [234]. Several natural compounds hold promise as gut microbiota modifiers and cancer prevention/treatment aids. Notably, many dietary polyphenols can beneficially alter the gut microbiota and directly act as antioxidants, synergistically enhancing probiotic effects [235]. Polyphenols such as RV and quercetin boost intestinal health by modulating Nrf2 and NF-κB, thus improving barrier function, repairing the gut lining, reducing inflammation, and regulating immunity. RV and quercetin modulate intestinal tight junction protein expression and distribution, thus raising trans-epithelial electrical resistance (TEER), according to reports. Therefore, they beneficially impact the gut barrier, helping to manage numerous diseases stemming from gut barrier dysfunction [236]. In diet-induced obese mice, oleuropein’s protection of metabolism and vasculature is linked to its immunomodulatory effects and improved gut barrier function by reducing dysbiosis [179]. A recent *in vivo* study highlighted that a bergamot polyphenol extract (BPE) with micronized albedo and pulp fibers (BMF) helps maintain gut microbiota health in animals fed an HFD. A four-week trial using a 50/50% BPE/BMF mixture in rats fed a HFD resulted in significantly better metabolic profiles and lower gut LPS levels, which are vital in cancer development [237,238], reducing MDA levels and leading lipoprotein size re-arrangement. This effect is related to improvements in gut microbiota composition, particularly through the modulation of the Gram-negative bacteria *Proteobacteria* and the dominant phyla *Firmicutes* and *Bacteroidetes* [110]. The findings suggest that the interaction between nutraceuticals, microbiota, and cancer is a highly promising field of research, and could offer new strategies for prevention and therapeutic support in oncology. Indeed, a complex interplay of various cell types with diverse metabolic states and shared metabolites creates the TME. The complex cellular structure produces a profile of available nutrients and reactive metabolites, including advanced glycation end-products. The literature analysis reveals that the receptor for advanced glycation end products (RAGE) and its ligands link energy metabolism (often disrupted by mitochondrial dysfunction) to immune responses (shaped by local microbiota), impacting tumor development. Investigating metabolic processes in cancer and immune cells reveals novel therapeutic strategies, potentially broadening immunotherapy’s reach and effectiveness. Targeting the RAGE and its ligands in immunotherapy shows promise for treating CRC, according to the literature review [239].

## 6. Limitations of Nutraceutical Supplementation: Current Challenges and Emerging Evidence-Based Strategies

Although nutraceuticals offer proven benefits, their widespread use in clinical practice is hindered by considerable limitations. Factors such as the poor bioavailability of active compounds, inconsistent dose-response, and insufficient clinical trial data hinder progress [240]. In addition, responses vary considerably between individuals due to genetic, gut microbiome, dietary, age-related, and health factors, complicating the determination of ideal dosages [241,242,243]. While exhibiting significant *in vitro* potential, the bioavailability of polyphenols (frequently researched for nutraceutical purposes) is low due to digestive breakdown and limited absorption in the upper gut. Primarily, only a small percentage of low molecular weight polyphenols (like glycosylated flavonoids) are absorbed in the upper small intestine, mainly the duodenum and jejunum. Most complex polyphenols bypass absorption until reaching the colon, undergoing microbial biotransformation into absorbable simpler metabolites (e.g., phenolic acids) [244]. In addition, a recent study demonstrated that innovative natural formulations significantly increased plasma metabolite levels in treated animals, overcoming the poor bioavailability of polyphenols and maximizing their beneficial effects [46]. Nutraceutical safety and efficacy, and their contribution to health, will improve if “bed-to-bench” issues are addressed.

## 7. Conclusions

This review summarizes results and evidence showing that Mediterranean nutraceuticals hold promise in cancer prevention and treatment. The antioxidant, anti-inflammatory, and pro-apoptotic properties of Mediterranean nutraceuticals, such as polyphenols and phytochemicals from *Citrus bergamia* Risso & Poiteau, *Cynara cardunculus*, *Olea europea* L., quercetin, resveratrol, and *Ferula communis* L., are promising as complementary treatments against cancer, providing a solid basis for considering them as effective adjuvants in conventional cancer therapies. Crucially, many nutraceuticals demonstrate dual dose-dependent effects; low concentrations act as antioxidants, and high concentrations induce selective cancer cell death via oxidative stress. This two-stage process holds significant promise for preventing and enhancing the effectiveness of cancer treatment: better outcomes and reduced side effects, particularly cardiotoxicity, may result from using them with standard cancer treatments. Furthermore, the interaction of natural compounds with the main molecular pathways involved in chronic inflammation (e.g., NF-κB, STAT3, PI3K/Akt) and oxidative stress (e.g., Nrf2/KEAP1, AMPK/SIRT1) highlights their potential in remodeling the TME. The active control of metastasis and chronic inflammation is suggested by the ability to adjust macrophage polarization, cytokine production, and the innate immune response. Developing validated anticancer therapies critically depends on integrating nutraceuticals, oncology, and pharmacology to optimize drug mechanisms, combinations, nutraceutical dosages (BPF, cynaropicrin, quercetin, RV, and ferutinin), and therapeutic translation. Despite this, natural compounds are not intended to replace conventional cancer treatments like chemotherapy, but rather to enhance them in a personalized approach for each patient. Low bioavailability, lack of extensive clinical trials, and inconsistent patient responses are delaying the clinical translation of these results. The efficacy of nutraceuticals in oncology requires confirmation through rigorous randomized controlled trials to implement positive preclinical results into proven therapeutic applications. Further *in vitro* and *in vivo* studies are needed to optimize bioavailability (e.g., formulation with nanocarriers), characterize pharmacokinetics, determine optimal dosages, and evaluate long-term safety. In closing, the emerging concept of onconutraceuticals represents an integrated paradigm that combines prevention, therapeutic support, and personalized medicine. In this context, the “Mediterranean” approach could offer a sustainable and evidence-based dietary-pharmacological model, useful not only for the management of neoplastic disease but also for the promotion of global health.

## Figures and Tables

**Table 1:** The Mediterranean Diet, nutraceuticals, and their biological properties.

Nutraceuticals	Bioactive Compounds	Main Sources	Biological Effects	References
*Citrus bergamia* Risso & Poiteau extract	Naringin, neohesperidin, neoeriocitrin, C-glucoside, flavanone O-glycosides, rhoifolin, 40-O-glucoside, neodiosmin, rhoifolin, poncirin limonene, linalool, linalyl acetate	Bergamot juice (BJ), bergamot essential oil (BEO)	Hypolipemic, hypoglycaemic anti-inflammatory, antioxidant, anticancer	[44,45,46,47]
*Cynara cardunculus* L. extract	Cynaropicrin (cyn), chlorogenic acid, dicaffeoylquinic acids, luteolin, inulin	Leaves, flowers, roots, by-products	Hepatoprotective, antioxidant, antimicrobial, antiobesity, chemopreventive	[48,49]
*Olea Europaea* L. extract	Hydroxytyrosol, oleuropein, tyrosol, oleic acid, omega-3, omega-6	Olive oil (OO), olive leaves, Olive mill wastewater (OMWW)	Antioxidant, cardioprotective, antitumoral, anti-inflammatory, anti-aging, antibacterial, prevention of metabolic disorders and chronic diseases	[50,51,52,53]
Quercetin	Flavonol (free and glycosylated forms)	Onions, apples, berries, broccoli, tea, cherries, tomatoes, asparagus, peas, grapes, coriander seeds	Antioxidant, anti-inflammatory, antiproliferative, antiviral, cardioprotective, antiaging, prevention of metabolic disorders, chronic diseases, platelet aggregation, lipid peroxidation, and capillary permeability, modulating the composition of the gut microbiota	[54,55]
Resveratrol	Trans-RV	Grapes skin, red wines, blueberry, cranberry, peanuts, bilberry	Antioxidant, anti-inflammatory, pro-apoptotic, antitumor, telomerase inhibition, improves gut barrier	[30,56,57,58]
*Ferula Communis* L. extract	Ferutinin, sesquiterpenes, ferulenol, ferulone A and B, flavonoids	Roots, rhizomes, latex	Selective estrogen receptor modulator (SERM-like), antiproliferative, dose-dependent estrogenic effect; antioxidant effects, antidiabetic, antimicrobial, cytotoxic actions	[59,60]

BJ: bergamot juice; BEO: bergamot essential oil; Cyn: cynaropicrin; OO: olive oil; OMWW: olive mill wastewater; RV: resveratrol; SERM-like: selective estrogen receptor modulator.

**Table 2:** Dose-dependent Action of Nutraceuticals.

Nutraceutical	Low Dose Effect (Protective/Antioxidant)	High Dose Effect (Pro-Oxidant/Anticancer)	Mechanism	References
Bergamot polyphenolic fraction	↓ MDA/ROS; ↑ SOD/GPX; ↓ 3-NT/LOX-1; ↑ protective effect.	Synergistic support in redox balance, not directly cytotoxic.	↓ peroxynitrite; ↑ antioxidant enzymes (cardiomyocytes, liver).	[108,109,110,111,113,128,143]
Cynaropicrin	↑ Nrf2/SOD/CAT/GSH-PX; antioxidant in A375, brain, liver tissues.	Inhibits TxR → ↑ ROS → apoptosis in cancer cells.	Activates antioxidant genes (GCL, HMOX-1), pro-apoptotic in melanoma, neural, liver cells.	[117,118,119]
Oleuropein	↑ SOD/GPX/GRX/CAT; ↓ ROS; protects membrane and ER integrity.	↑ ROS, mitochondrial dysfunction, cyt C release; apoptotic in MCF-7, HepG2, HEY cancer cells.	Dose- and cell-type specific redox modulation.	[124,125]
Quercetin	Activates Nrf2; ↑ GSH/SOD/GPX; inhibits Topo II; protection from Dox cardiotoxicity.	↑ ROS; DNA damage; cyt C release; apoptosis via intrinsic/extrinsic pathways.	Caspase cascade, KEAP1 oxidation, ARE activation, Topo II inhibition.	[132]
Resveratrol	↑ SIRT1/AMPK/FOXO3a, antioxidant genes; prevents Dox toxicity.	↑ ROS; mitochondrial permeability transition pore (mPTP) opening; apoptosis through caspase activation.	Mitochondrial depolarization; dual effect depending on dose and exposure time.	[133,134,135]
Ferutinin	Antioxidant, phytoestrogenic; ↓ ROS/MDA; protects H9c2 and neural cells.	↑ ROS, ↑ Bax, cyt C; apoptosis in MCF-7, MDA-MB-231 BC cells.	SERM-like; mitochondrial apoptosis; cell-selective effects.	[138,139,140,141,142]

The arrows indicate decrease (↓) and increase (↑), and progression (→), respectively; MDA: malondialdehyde; ROS: reactive oxygen species; SOD: superoxide dismutase; GPX: glutathione peroxidase; 3-NT: 3-nitrotyrosine; LOX-1: lectin-like oxidized low-density lipoprotein receptor-1; Nrf2: nuclear erythroid 2-related factor; CAT: catalase; GSH-PX: glutathione peroxidase; TxR: thioredoxin reductase; GCL: glutamate-cysteine ligase; HMOX-1: heme oxygenase; GRX: glutathione reductase; Cyt C: cytochrome C; GSH: glutathione; Topo II: topoisomerase II; Dox: doxorubicin; DNA: deoxyribonucleic acid; KEAP1: kelch-like ECH-associated protein 1; ARE: antioxidant response element; SIRT1: silent mating type information regulation 2 homolog 1; AMPK: AMP-activated protein kinase; FOXO3a: forkhead transcription factor O subfamily member 3a; mPTP: mitochondrial permeability transition pore; BC: breast cancer; SERM-like: selective estrogen receptor modulator-like.

**Table 3:** Effects of Natural Compounds on Cell Cycle Regulation.

Natural Compound/Plant	Cell Cycle Phase	Molecular Mechanism	Cell Type	References
*Citrus bergamia*	G1/S	Silent mating type information regulation 2 homolog 2 (SIRT2)/Akt/p53 ↑ apoptosis	THP-1, SH-SY5Y	[196,197,198]
Cynaropicrin	G2/M	↓ Pyruvate kinase M2 (PKM2)/PARP	CAL-62, 8505C, SW1736, A549	[199,200]
Oleuropein	G1/S	↑ p21, p53, CKIs	MCF-7, MDA-MB-231, MDA-MB-468	[201,202,203]
Resveratrol	S	↑ p53/p21/p27/Bax/p53 upregulated modulator of apoptosis (PUMA) ↓ cyclins D/E ↓ cyclin-dependent kinase 2, 4, 6 (CDK2/4/6)	C-3, LNCaP, A431, T47D	[204,205,206,207,208]
Quercetin	G1/G2/M	↑ p21/p27 ↓ CDK2/6, cyclins A/E/D ↓ Rb phosphorylation	HOS, OSCC, T47D, A375, p39, YD10B, YD38, OSCC, KON	[209,210,211,212]
Ferutinin	↓ G1 and restoring cell cycle	↑ apoptosis ↑ protective effect	H9c2	[138,140,213]

The arrows indicate decrease (↓) and increase (↑), respectively. SIRT2: silent mating type information regulation 2 homolog 2; PKM2: pyruvate kinase M2; PARP: poly-ADP-ribose-polymerase; p53: tumor protein p53; CKIs: CDK inhibitors; Bax: BCL2 associated X, apoptosis regulator; PUMA: p53 upregulated modulator of apoptosis; CDK: cyclin-dependent kinase; Rb: retinoblastoma protein.

## Abbreviations

8-oxodG	8-oxo-2′-deoxyguanosine
8-OHdG	8-hydroxy-2′-deoxyguanosine
3-NT	3-nitrotyrosine
AGEs	advanced glycation end products
Akt	protein kinase B
AMPK	AMP-activated protein kinase
APAF1	apoptotic protease-activating factor 1
ARE	antioxidant response element
ATM	ataxia-telangiectasia mutated
ATP	Adenosine Triphosphate
BAD	BCL2 associated agonist of cell death
BAT	brown adipose tissue
Bax	BCL2 Associated X, Apoptosis Regulator
BC	breast cancer
BCL2L11	B-cell lymphoma 2 (Bcl-2)-like protein 11
BEO	bergamot essential oil
Birc5	Baculoviral Inhibitor of Apoptosis Repeat-containing 5
BMF	albedo and pulp-derived micronized fibers
BJ	bergamot juice
BPE	bergamot polyphenol extract
BPF	bergamot polyphenolic fraction
BRAF	B-Raf proto-oncogene serine/threonine kinase
c-Met	mesenchymal–epithelial transition factor
c-myc	cellular myelocytomatosis oncogene
CAFs	cancer-associated fibroblasts
CAT	catalase
CDK	cyclin-dependent kinase
CDKs	cyclin-dependent kinases
Chk1	checkpoint kinases 1
Chk2	checkpoint kinases 2
CIC	chemotherapy-induced cardiac damage
CIN	chromosomal instability
CKIs	CDK inhibitors
CLRs	C-type lectin receptors
COX-1	cyclooxygenases 1
COX-2	cyclooxygenases 2
CRC	colorectal carcinoma
CRP	C reactive protein
CSCs	cancer stem cells
CVD	cardiovascular disease
Cyn	cynaropicrin
Cyt C	cytochrome C
Daun	daunorubicin
DCs	dendritic cells
DISCs	death-inducing signaling complexes
DM	diabetes mellitus
DNA	DeoxyriboNucleic Acid
DNMTs	DNA methyltransferases
DSBs	double-strand breaks
Dox	doxorubicin
E. coli	Escherichia coli
EC	endometrial cancer
ECM	extracellular matrix
ECs	endothelial cells
eCSCs	endogenous cardiac stem cells
EFSA	The European Food Safety Authority
EGFR	epidermal growth factor receptor
EMT	Epithelial–Mesenchymal Transition
eNOS	endothelial nitric oxide synthase
ER	endoplasmic reticulum
ERK	extracellular signal-regulated kinase
ERK1/2	extracellular signal-regulated kinase ½
ERα	Estrogen receptor alpha
ERβ	Estrogen receptor beta
EVOO	extra virgin olive oil
FADD	Fas-Associated Death Domain Protein
FOXO	Forkhead box transcription factors
FOXO3a	Forkhead Transcription Factor O Subfamily Member 3a
FRE	Flavonoid-Rich Extract
GCL	glutamate-cysteine ligase
GGT	gamma-glutamyl transferase
GPX	glutathione peroxidase
GRX	glutathione reductase
GSH-PX	Glutathione Peroxidase
GSH	glutathione
GSK-3β	glycogen synthase kinase 3 beta
GST	glutathione transferase
GSTs	glutathione S-transferases
H. pylori	Helicobacter pylori
H_2_O_2_	Hydrogen Peroxide
H9c2	rat embryonic cardiac myoblast
HBV	Hepatitis B Virus
HCC	hepatocellular carcinoma
HCV	Hepatitis C virus
HDACs	histone deacetylases
HDL	high density lipoprotein
HFD	high-fat diets
HIF1-α	hypoxia-inducible factor 1-alpha
HLD	hyperlipidemic diet
HMOX-1	heme oxygenase
HO-1	heme oxygenase-1
HOCl	hypochlorous acid
HPV	Human Papillomavirus
HRAS	Harvey Rat sarcoma virus
HRT	hormone therapy
HUVECs	Human Umbilical Vein Endothelial Cells
I/R	ischemia–reperfusion
IARC	International Agency for Research on Cancer
ICAM-1	intercellular adhesion molecule 1
ICAM-2	intercellular adhesion molecule 2
IFN-γ	interferon-gamma
IL-10	Interleukin-10
IL-10R2	Interleukin-10 Receptor Subunit 2
IL-12	Interleukin-12
IL-13	interleukin-13
IL-1R	interleukin-1 receptor
IL-1α	Interleukin-1 alpha
IL-1β	Interleukin-1 beta
IL-22R1	Interleukin-22 Receptor Subunit 1
IL-23	interleukin-23
IL-4	interleukin-4
IL-6	interleukin-6
IL-6R	interleukin-6 receptor
IL-8	interleukin-8
iNOs	Inducible nitric oxide synthase
ITM	intratumoral microbiota
JAK-STAT	Janus kinase (JAK)/signal transducer and activator of transcription (STAT)
JAK1/TYK2	Janus kinase 1 and Tyrosine kinase 2
JNK-1	c-Jun N-terminal kinase 1
JNK	c-Jun amino-terminal kinase
KEAP1	Kelch-like ECH-associated protein 1
KRAS	Kirsten rat sarcoma viral oncogene homolog
LDL	low density lipoprotein
LOX-1	Lectin-like oxidized low-density lipoprotein receptor-1
LOX	lipoxygenase
LPS	lipopolysaccharide
LTB4	leukotriene B4
MAPK	Mitogen-Activated Protein Kinase
MDA	Malondialdehyde
MDSCs	myeloid-derived suppressor cells
MedDiet	Mediterranean Diet
MIP-2	Macrophage-inflammatory protein 2
MKP-1	Mitogen-activated protein kinase phosphatase 1
MOMP	mitochondrial outer membrane permeabilization
MS	metabolic syndrome
mTOR	mammalian target of rapamycin
mTORC1	mammalian target of rapamycin complex 1
MUFAs	monounsaturated fatty acids
NADPH	nicotinamide adenine dinucleotide phosphate
NAFLD	Non-alcoholic fatty liver disease
NF-κβ	Nuclear factor kappa-light-chain-enhancer of activated B cells
NGS	next-generation sequencing
NK	natural killer
NLRs	NOD-like receptors
NOTCH	Notch signaling pathway
NQO1	NAD(P)H quinone oxidoreductase 1
NRAS	Neuroblastoma RAS viral oncogene homolog
Nrf2	nuclear erythroid 2-related factor
O_2_^−^	superoxide anion
O_2_	dioxygen
OMWW	Olive Mill Wastewater
OxLDL	oxidized low-density lipoprotein
P53	tumor protein p53
PAF	platelet-activating factor
PAMPs	pathogen-associated molecular patterns
PARP-1	poly (ADP-ribose) polymerase-1.
PARP	poly-ADP-ribose-polymerase
PC	pancreatic cancer
PI3K	phosphatidylinositol 3-kinase
PKB	protein-kinase B
PKC	protein kinase C
PKD1	Polycystin 1, Transient Receptor Potential Channel Interacting
PKM2	Pyruvate kinase M2
PLA2	phospholipase A2
PLK1	polo-like kinase 1
PMN-MDSC	polymorphonuclear myeloid-derived suppressor cells
PPARγ	peroxisome proliferator-activated receptor-gamma
PRX	peroxiredoxin
PTEN	Phosphatase and tensin homolog
PTMs	post-translational modifications
PTP1B	Tyrosine-protein phosphatase non-receptor type 1
PTPs	Protein Tyrosine Phosphatases
PUFAs	polyunsaturated fatty acids
PUMA	p53 upregulated modulator of apoptosis
RAGE	Receptor for advanced glycation end products
RAS	Rat sarcoma virus
Rb	Retinoblastoma protein
Ref-1	Redox Effector Factor-1
RLRs	RIG-I-like receptors
RNS	Reactive nitrogen species
ROS	Reactive oxygen species
RV	Resveratrol
SAC	spindle assembly checkpoint
SERM	selective estrogen receptor modulator
SIRT1	silent mating type information regulation 2 homolog 1
SIRT2	Silent mating type information regulation 2 homolog 2
SFRP4	secreted frizzled-related protein 4
SOD	superoxide dismutase
STAT	Signal Transducer and Activator of Transcription
STAT3	Signal Transducer and Activator of Transcription 3
T2DM	type 2 diabetes mellitus
TAMs	tumor-associated macrophages
TEER	trans-epithelial electrical resistance
TFs	transcription factors
TGF-β	Transforming growth factor beta
Th2	helper T 2 cells
TLR	Toll-like receptor
TME	tumor microenvironment
TNF-α	Tumor Necrosis Factor Alpha
TNFR-1	tumor necrosis factor receptor 1
Topo II	topoisomerase II
TRAIL-R1/2	tumor necrosis factor-related apoptosis-inducing ligand receptors
Tregs	regulatory T-cells
TRX	thyroxine
TXNRD1	thioredoxin reductase-1
TxR	thioredoxin reductase
UV	Ultraviolet radiation
VEGF	Vascular Endothelial Growth Factor
VOO	virgin olive oil
WAT	white adipose tissue
WD SW	High-fat Western diet-fed
Wnt	Wingless-related integration site
XO	xanthine oxidase
ZEB1/2	Zinc finger E-box binding homeobox 1 and Zinc finger E-box binding homeobox 2
ZFP57	zinc finger protein 57
